# Influence of *N*‐Functionalization of Amiridine on the Biological Activity of Its Conjugates as Multitarget Agents for Potential Treatment of Alzheimer's Disease

**DOI:** 10.1002/cmdc.70392

**Published:** 2026-07-24

**Authors:** Galina F. Makhaeva, Tatiana Y. Astakhova, Maria V. Grishchenko, Tatiana S. Shteinberg, Nadezhda V. Kovaleva, Natalia P. Boltneva, Elena V. Rudakova, Pavel G. Pronkin, Elena N. Timokhina, Eugene V. Radchenko, Evgeny V. Shchegolkov, Yanina V. Burgart, Victor I. Saloutin, Valery N. Charushin, Rudy J. Richardson

**Affiliations:** ^1^ Institute of Physiologically Active Compounds at Federal Research Center of Problems of Chemical Physics and Medicinal Chemistry Russian Academy of Sciences Chernogolovka Russia; ^2^ Emanuel Institute of Biochemical Physics Russian Academy of Sciences Moscow Russia; ^3^ Postovsky Institute of Organic Synthesis Ural Branch of Russian Academy of Sciences Ekaterinburg Russia; ^4^ Department of Chemistry Lomonosov Moscow State University Moscow Russia; ^5^ Department of Environmental Health Sciences University of Michigan Ann Arbor Michigan USA; ^6^ Department of Neurology University of Michigan Ann Arbor Michigan USA; ^7^ Center of Computational Medicine and Bioinformatics University of Michigan Ann Arbor Michigan USA; ^8^ Michigan Institute for Computational Discovery and Engineering University of Michigan Ann Arbor Michigan USA; ^9^ Michigan Institute for Data and AI in Society University of Michigan Ann Arbor Michigan USA

**Keywords:** Alzheimer's disease, amiridine, multitarget agents, *N*‐functionalization, salicylimine/amine

## Abstract

The goal of this mechanistic study was to elucidate the influence of *N*‐functionalization of amiridine on the biological properties of its conjugates. We synthesized conjugates of amiridine and salicylimine/amine **9, 10** with linkers containing an *N*‐acyl group at the amiridine fragment as analogs of our previously obtained hybrids **1, 2** containing an *N*‐aminohexamethylene spacer. A comparative study of selected biological activities of *N*‐acylalkylene conjugates **9, 10** with conjugates **1, 2** showed substantial decreases in anti‐butyrylcholinesterase (BChE) activity (35‐fold for the imine and 8‐fold for the amine), loss of anti‐acetylcholinesterase (AChE) activity, and disappearance of the ability to block amyloid beta (1–42) (Aβ_42_) self‐aggregation. Similar effects were observed for the model compound *N*‐hexylamiridine **3** and its *N*‐acyl derivative **11**. However, *N*‐acyl functionalization at the amiridine pharmacophore did not reduce the ability of the conjugates to block AChE‐induced β‐amyloid aggregation and only slightly decreased their antiradical activity, maintaining the higher activity of amines compared to imines. These effects were consistent with results from quantum mechanical calculations and molecular docking, showing that *N*‐acylation of amiridine decreased the proton affinity of its endocyclic N‐atom. Consequently, the amiridine pharmacophore was essentially unprotonated, thereby diminishing the anticholinesterase activity of conjugates **9** and **10** and their ability to block Aβ_42_ self‐aggregation.

## Graphical abstract

New conjugates of amiridine and salicylimine/amine with an *N*‐acylalkylene spacer were synthesized. *N*‐acylation of amiridine conjugates leads to a decrease in their protonation, resulting in a substantial reduction in anticholinesterase activity and relative inability to block Aβ_42_ self‐aggregation compared to previously studied analogs with an *N*‐aminohexamethylene spacer.

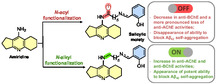

## 1 Introduction

The purpose of the present work was to gain mechanistic insight into the nature of the influence of *N*‐functionalization of amiridine on the biological properties of its conjugates that have relevance to the design of therapeutic agents for Alzheimer's disease (AD). In order to show how the molecular features of *N*‐acylation worsen the biological profile of conjugates based on amiridine, we compared biological activities of previously studied conjugates containing an *N*‐alkylene spacer with new *N*‐acyl analogs. Thus, our purpose was not to discover more potent conjugates but to discover why certain conjugates had diminished potency. However, we expect that the knowledge gained from this investigation would also have fruitful applications to the discovery of improved anti‐AD drugs in future studies.

AD exhibits multifactorial etiology. The main factors characterizing the pathogenesis of AD include disruption of neurotransmitter systems, deposition of aberrant proteins (beta‐amyloid and tau protein), oxidative stress, mitochondrial dysfunction, loss of synapses, and death of nerve cells, especially cholinergic neurons [1, 2, 3]. Taking into account the multifaceted nature of AD, a promising approach to the development of drugs for its treatment is the creation of multitarget agents capable of the simultaneous suppression of two or more biological effectors responsible for the pathogenesis of the disease [1, 4, 5, 6, 7, 8].

The strategy of rational design of multitargeted drugs is based on the linkage (through a spacer) of two pharmacophores exhibiting activity against at least two different molecular targets. Given the cholinergic deficiency characteristic of AD, known cholinesterase (ChE) inhibitors are often employed as the first pharmacophore to boost acetylcholine levels, thereby providing the symptomatic cognitive‐stimulating properties of the new molecule. The introduction of an appropriate second pharmacophore can enable the conjugate to confer additional neuroprotective and disease‐modifying properties [1, 5, 9, 10, 11, 12, 13].

The use of the anticholinesterase drug amiridine in the treatment of senile dementia and AD [14, 15, 16, 17, 18, 19] makes it a promising candidate for the development of multitargeted drugs. Structurally, this drug is a bicycloalkane derivative of aminopyridine and resembles tacrine, but amiridine has significantly fewer cholinergic side effects, does not exhibit hepatotoxicity, and has a wider therapeutic window [20, 21]. Due to its good permeability through the blood‐brain barrier, amiridine quickly enters the brain and accumulates primarily in the cerebral cortex and hippocampus—the structures most susceptible to damage in AD [20]. These factors make amiridine an attractive anticholinesterase pharmacophore for the development of multitarget drugs for the treatment of AD.

Considering the low reactivity of amiridine [22, 23], its functionalization is a rather difficult task. Our team has pioneered the use of amiridine as a basis for multifunctional conjugates. To introduce the spacer, we modified the amiridine exocyclic amino group using highly reactive chloroalkanoic acid chlorides [24, 25] and thiophosgene [26]. Conjugation with a second pharmacophore was carried out by interaction with the reactive group of the introduced spacer. In this manner, we have obtained several classes of amiridine conjugates with linkers containing *N*‐acyl‐ or *N*‐thiourea groups at the amiridine moiety [24, 25, 26, 27, 28, 29].

The synthesized conjugates exhibited a range of biological properties relevant to AD, including anticholinesterase activity, inhibition of β‐amyloid aggregation, and antioxidant activity. The pharmacological activity of the hybrid compounds was determined by both the second pharmacophore and the structure of the spacer. However, a characteristic feature of conjugates with an *N*‐acyl‐ or *N*‐thiourea group at the amiridine moiety was decreased inhibitory activity against both ChEs, particularly AChE, compared to the parent amiridine. We hypothesized that this effect could be due to the presence of an acyl‐ or thioacyl‐containing spacer fragment directly adjacent to amiridine as a consequence of its functionalization.

It is known that acylation at the external nitrogen of amiridine leads to a decrease in the p*K*_a_ of the acylated derivative [22, 23]. Moreover, our previous computational studies using empirical (ChemAxon Marvin) and QM (Schrödinger Jaguar) methods showed that the estimated p*K*_a_ of the amiridine fragment in the conjugates was significantly decreased relative to the parent amiridine [24]. These experimental and computational results indicated that under the conditions of the anti‐ChE assays, the amiridine ligand would be largely unprotonated, and molecular docking simulations predicted that the unprotonated species would have decreased binding affinity toward ChEs relative to the protonated species [24].

To avoid the presence of an acyl moiety attached to amiridine in conjugates, we proposed an alternative conjugation strategy based on the use of its chloro derivative in substitution reactions with diaminoalkanes. This approach yielded the first new type of conjugates in which the amiridine molecule was linked to a second pharmacophore via an aminoalkylene spacer of varying length. Salicylic derivatives [30], aminomethylidene derivatives of trifluoroacetoacetic ester [31], and vitamin B_6_ derivatives [32] were used as the second pharmacophore. Indeed, the new conjugates showed high anti‐ChE activity (exceeding the activity of the amiridine pharmacophore), effectively blocked AChE‐induced and self‐aggregation of β‐amyloid, and exhibited high radical‐scavenging activity (as salicylic and vitamin B_6_ derivatives).

**FIGURE 1:**
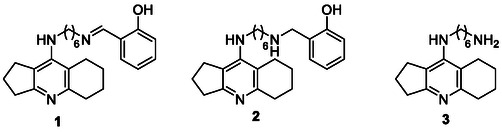
Amiridine conjugates with salicylimine/amine linked by an aminohexamethylene spacer **1**, **2,** and an aminohexyl derivative of amiridine **3** [30].

In the present work, to test our mechanistic hypothesis about the critical role of the carbonyl group at amiridine on the biological activity of its conjugates, we synthesized *N*‐acyl analogs of previously studied conjugates with salicylimine/amine combined with an aminohexamethylene spacer (**1**, **2**) (Figure 1). Compounds **1** and **2** exhibited potent anti‐ChE activity, especially toward BChE, antiaggregatory properties toward β‐amyloid, and high ABTS‐scavenging activity. In addition, an *N*‐acyl analog of the model *N*‐hexylamiridine **3** was synthesized [30]. A spectrum of biological activity of newly synthesized compounds was studied in comparison with corresponding activities of compounds **1–3.**

## 2 Results and Discussion

### 2.1 Synthesis of Target Compounds

**SCHEME 1:**
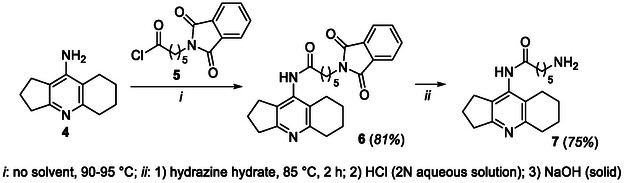
Synthesis of 6‐amino‐*N*‐(2,3,5,6,7,8‐hexahydro‐1*H*‐cyclopenta[*b*]quinolin‐9‐yl)hexanamide **7**.

At the first step of the synthesis of target conjugates containing an *N*‐acyl fragment, amiridine **4** was acylated with 6‐(1,3‐dioxoisoindolin‐2‐yl)hexanoic acid chloride **5** in the absence of a solvent at 90 °C–95 °C, yielding compound **6** (Scheme 1). It is worth noting that the acylation reaction did not proceed at lower temperatures (25 °C–85  °C). Removal of the phthalimide protecting group from compound **6** by sequential treatment with hydrazine hydrate, aqueous HCl, and KOH afforded 6‐amino‐*N*‐(2,3,5,6,7,8‐hexahydro‐1*H*‐cyclopenta[*b*]quinolin‐9‐yl)hexanamide **7** with a free terminal NH_2_‐group, suitable for further transformations.

**SCHEME 2:**
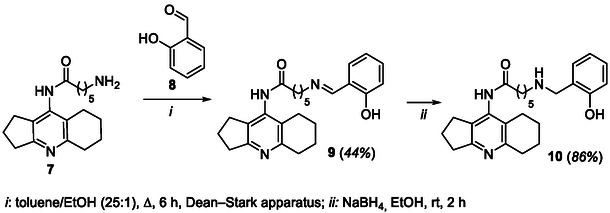
Synthesis of conjugates **9**, **10**.

Condensation of compound **7** at the amino group with salicylic aldehyde **8** in a toluene/EtOH mixture (25:1) with azeotropic distillation of water yielded imine **9**, by reduction of which with NaBH_4_ in EtOH amine **10** was synthesized (Scheme 2).

**SCHEME 3:**
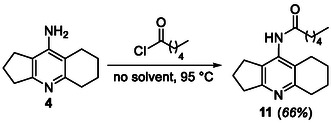
Synthesis of *N*‐(2,3,5,6,7,8‐hexahydro‐1*H*‐cyclopenta[*b*]quinolin‐9‐yl)hexanamide **11**.

In addition, to assess the influence of the *N*‐acyl group on the activity of amiridine, we synthesized a model derivative, *N*‐(2,3,5,6,7,8‐hexahydro‐1*H*‐cyclopenta[*b*]quinolin‐9‐yl)hexanamide **11**, as a result of acylation of amiridine **4** with hexanoyl chloride upon heating to 95 °C in the absence of a solvent (Scheme 3).

The structure of compounds **9**–**11** was confirmed by ^1^H and ^13^C NMR spectroscopy, IR spectroscopy, and elemental analysis. Thus, the IR spectra of all compounds exhibited an absorption band corresponding to the C=O group at *ν* 1656–1661 cm^−1^. In the ^13^C NMR spectra of **9**–**11**, the characteristic signal of the C=O carbon atom was observed at *δ *∼ 170 ppm.

### 2.2 Biological Activities

A comparative study of the biological activity of synthesized conjugates with an *N*‐acylalkylene spacer **9, 10** versus their *N*‐alkylene analogs **1, 2** [30] was conducted. The study included an assessment of the esterase profile (inhibitory activity toward the cholinergic targets AChE and BChE as well as the structurally related enzyme carboxylesterase (CES), which hydrolyzes numerous ester‐containing drugs and is considered an off‐target for anti‐AD agents [33]), determination of the ability to block AChE‐induced aggregation of β‐amyloid (by displacement of propidium from the *Electrophorus electricus* (*Ee*) AChE peripheral anionic site (PAS)), inhibition of Aβ_42_ self‐aggregation, and evaluation of the primary antioxidant activity in the 2,2^′^‐azino‐bis(3‐ethylbenzothiazoline‐6‐sulfonic acid (ABTS) and ferric‐reducing antioxidant power (FRAP) tests. The results are shown in Table 1.

**TABLE 1:** Esterase profiles of the compounds **1–3, 9–11**, their ability to displace propidium from the PAS of *Electrophorus electricus* AChE (*Ee*AChE), inhibition of Aβ_42_ self‐aggregation, and antioxidant activity in the ABTS test.

Compound	Inhibitory activity against AChE, BChE and CES			Propidium displacement, %^c^	Inhibition of Aβ_42_ self‐aggregation, %^d^	ABTS^•+^‐scavenging activity	
	AChE, IC_50_, μM^a^ or %^b^	BChE, IC_50_, μM^a^	CES, %^b^			TEAC^e^	IC_50_, μM
**1** f	3.25 ± 0.08	0.0620 ± 0.0060	12.4 ± 1.2	11.7 ± 0.8	59.1 ± 4.7	0.64 ± 0.03	28.2 ± 1.3
**2** f	1.19 ± 0.05	0.0294 ± 0.0006	18.6 ± 0.1	15.1 ± 1.2	79.2 ± 5.5	1.48 ± 0.07	9.5 ± 0.6
**3** f	4.30 ± 0.24	0.262 ± 0.008	5.5 ± 0.4	16.1 ± 1.1	46.4 ± 2.7	n.а.	n.d.
**9**	5.1% ± 0.9%	2.16 ± 0.07	3.7 ± 0.1	10.3 ± 0.7	n.а.	0.28 ± 0.02	74.5 ± 4.6
**10**	27.7% ± 1.3%	0.231 ± 0.007	20.7 ± 0.1	13.7 ± 0.9	n.а.	1.33 ± 0.05	11.7 ± 0.6
**11**	2.9% ± 0.4%	4.12 ± 0.28	2.6 ± 0.2	n.а.	n.а.	n.а.	n.d.
Amiridine	4.44 ± 0.36	0.272 ± 0.015	2.7 ± 0.5	12.2 ± 0.9	6.4 ± 0.5	0.040 ± 0.003	n.d.
BNPP	n.а.	n.а.	96.1 ± 0.6^g^	n.d.	n.d.	n.d.	n.d.
Myricetin	n.d.	n.d.	n.d.	n.d.	74.7 ± 5.2	n.d.	n.d.
Donepezil	0.040 ± 0.004	19.2 ± 3.0	n.а.	11.9 ± 0.9	n.d.	n.d.	n.d.
Trolox	n.d.	n.d.	n.d.	n.d.	n.d.	1.0	20.1 ± 0.9

Abbreviations: n.a., not active; n.d., not determined.

a For active compounds, AChE and BChE inhibition is presented as IC_50_ ± SEM μM, *n* = 3.

b AChE, BChE, and CES inhibition correspond to % inhibition at 20 μM.

c Propidium displacement from the *Ee*AChE PAS at 20 µM compound concentration.

d Inhibition of Aβ_42_ (50 µM) self‐aggregation by the tested compound at 100 µM concentration.

e TEAC (Trolox equivalent antioxidant capacity) was determined from the ratio of the slopes of the concentration‐response curves, test compound/Trolox.

f data for compounds **1–3** are taken from the work [30].

g bis(4‐nitrophenyl) phosphate (BNPP) IC_50_ CES = 1.80 ± 0.11 μM. All values are presented as mean ± SEM, *n* = 3.

#### 2.2.1 Inhibition of AChE, BChE, and CES

Conjugates of amiridine and salicylic derivatives with an *N*‐alkylene spacer (**1, 2**) were effective ChE inhibitors: inhibition of AChE – IC_50_ 1.19 and 3.25 μM, inhibition of BChE – IC_50_ 0.0294 and 0.062 μM with marked selectivity for BChE [30]. Replacement of the *N*‐alkylene spacer in conjugates **1** and **2** by the *N*‐acylalkylene spacer led to a substantial decrease in the inhibitory activity of conjugates **9** and **10** against BChE (34.8 times for the salicylimine and 7.9 times for the salicylamine) (Table 1) and to an even greater decrease in anti‐AChE activity: from micromolar IC_50_ to 5.1% and 27.7% at a concentration of 20 μM for conjugates with a salicylimine **9** and salicylamine **10**, respectively.

A study of model compounds **3** and **11** showed that *N*‐alkylation of amiridine had virtually no effect on its anti‐AChE and anti‐BChE activity (**3**), whereas its *N*‐acylation (**11**) reduced anti‐BChE activity by 15 times and virtually eliminated anti‐AChE activity to 2.9% ± 0.4% at 20 μM.

All synthesized conjugates (both with an *N*‐alkylene and *N*‐acylalkylene spacer) only weakly inhibited CES. Low potency against CES is considered a positive property of the compounds because this would be expected to reduce the likelihood of undesirable drug–drug interactions in the case of the use of the conjugates as potential therapeutic agents for AD treatment.

#### 2.2.2 Kinetic Studies of AChE and BChE Inhibition

**FIGURE 2:**
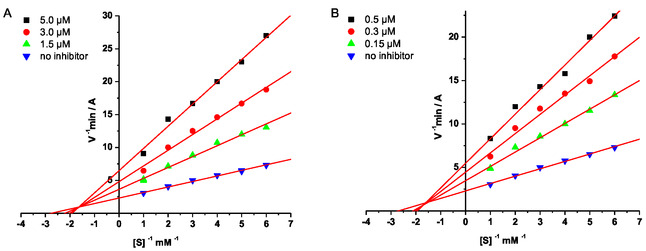
Steady state BChE inhibition by compounds **9** (A) and **10** (B).

The mechanism of BChE inhibition by the conjugates was studied using new conjugates **9, 10** with an *N*‐acylalkylene spacer. Lineweaver–Burk plots of the enzyme kinetics results are shown below (Figure 2). Analysis of the kinetics plots demonstrated changes in both *K* *_m_* and *V*_max_ values—a result consistent with a mixed type of inhibition as it was shown previously for conjugates **1**, **2** with *N*‐alkylene spacer [30]. The calculated inhibition constants are *K*_*i*_ = 1.72 ± 0.06 µM (competitive component) and α*K* *_i_* = 2.83 ± 0.09 µM (noncompetitive component) for compound **9;** *K*_*i*_ = 0.31 ± 0.02 µM and α*K*_*i*_ = 0.43 ± 0.03 µM for compound **10.**

#### 2.2.3 Propidium Displacement

To evaluate the synthesized compounds **9–11** as potential inhibitors of the proaggregating activity of AChE, we used a fluorescence method to determine the ability of the compounds to competitively displace propidium iodide, a selective ligand of the PAS of AChE responsible for binding to β‐amyloid, which effectively inhibits its AChE‐induced aggregation [34].

All the studied amiridine conjugates **9** and **10** (Table 1) as well as conjugates **1**, **2** at a concentration of 20 μM displaced propidium from the *Ee*AChE PAS at a level generally comparable to the reference compound donepezil (11.9% ± 0.9%) and parent compound amiridine (12.2% ± 0.9%).

Therefore, replacement of the *N*‐alkylene spacer by the *N*‐acylalkylene one had little effect on the ability of the conjugates to displace propidium from the AChE PAS, whereas conjugates **2, 10** with a salicylamine fragment in both cases were somewhat more active than their imine analogs **1, 9**. In contrast, a notable difference in this ability was observed between model compounds **3** and **11**.

#### 2.2.4 Inhibition of Aβ_42_ Self‐Aggregation

The ability of compounds **9–11** to inhibit Aβ_42_ self‐aggregation was studied using minor modifications of a fluorescence assay with Thioflavin T (ThT) [35, 36, 37].

Replacement of the *N*‐alkylene spacer by the *N*‐acylalkylene one led to disappearance of the ability of compounds **9–11** to block Aβ_42_ self‐aggregation (Table 1). The effect was very pronounced, considering that conjugates with the *N*‐alkylene spacer **1, 2** were effective antiaggregants (59.1% and 79.2% at 100 μM, respectively), compound **2** exceeded the activity of myricetin (74.7% ± 5.2%). It should be noted that we previously observed the absence of inhibition of Aβ_42_ self‐aggregation for bis‐*N*‐acyl‐amiridines [38].

#### 2.2.5 Antioxidant Activity

The primary antioxidant activity of the compounds **9–11** was evaluated using two standard assays. One is the FRAP test [39], which evaluates the ferric‐reducing activity of the conjugates. The second method is the ABTS assay, which measures radical‐scavenging activity [40]. Trolox was used as the reference antioxidant. The results showed that all synthesized conjugates did not exhibit ferric‐reducing activity in the FRAP test as was shown earlier [30].

The results of the ABTS assay (Table 1) demonstrated a more pronounced effect on activity with the presence of an amine (conjugates of salicylamine **2, 10**) or imine (conjugates of salicylimine **1, 9**) moiety in the molecule compared to the effect of *N*‐acylation of amiridine. Сonjugates of salicylamine **2** and **10** demonstrated high antiradical activity exceeding that of the standard antioxidant Trolox (TEAC 1.48 ± 0.07 and 1.33 ± 0.05). Substitution of the salicylamine pharmacophore with salicylimine led to a decrease in radical‐scavenging activity, with this effect being more pronounced for conjugates with an *N*‐acylalkylene linker (TEAC 0.64 ± 0.03 for **1** and 0.28 ± 0.02 for **9**).

#### 2.2.6 Molecular Docking to AChE and BChE

The protonation states of the investigated compounds **1–3** and **9–11** were assigned based on previous studies [30], QM calculations, and known experimental p*K*_a_ values of amiridine derivatives with *N*‐acetyl (p*K*_a_ = 5.7) and *N*‐methyl (p*K*_a_ = 9.5) functions at the exocyclic nitrogen atom [22, 23].

It was shown that *N*‐acylation of amiridine led to a substantial decrease in the proton affinity (PA) of its endocyclic *N*‐atom. The PA values of the endocyclic *N*‐atom in the amiridine moiety were found to be practically the same for all compounds with the same type of amiridine functionalization. Based on this, it was suggested that in *N*‐acylalkylene‐containing compounds **9**–**11**, the amiridine pharmacophore was neutral, whereas compounds **1–3** with an *N*‐alkylene fragment were protonated at the endocyclic nitrogen atom of amiridine. The protonation state of the second pharmacophore was chosen as it was done in previous studies [32]. Namely, the imine nitrogen was assumed to be neutral, while the amine nitrogen was assumed to be protonated.

**FIGURE 3:**
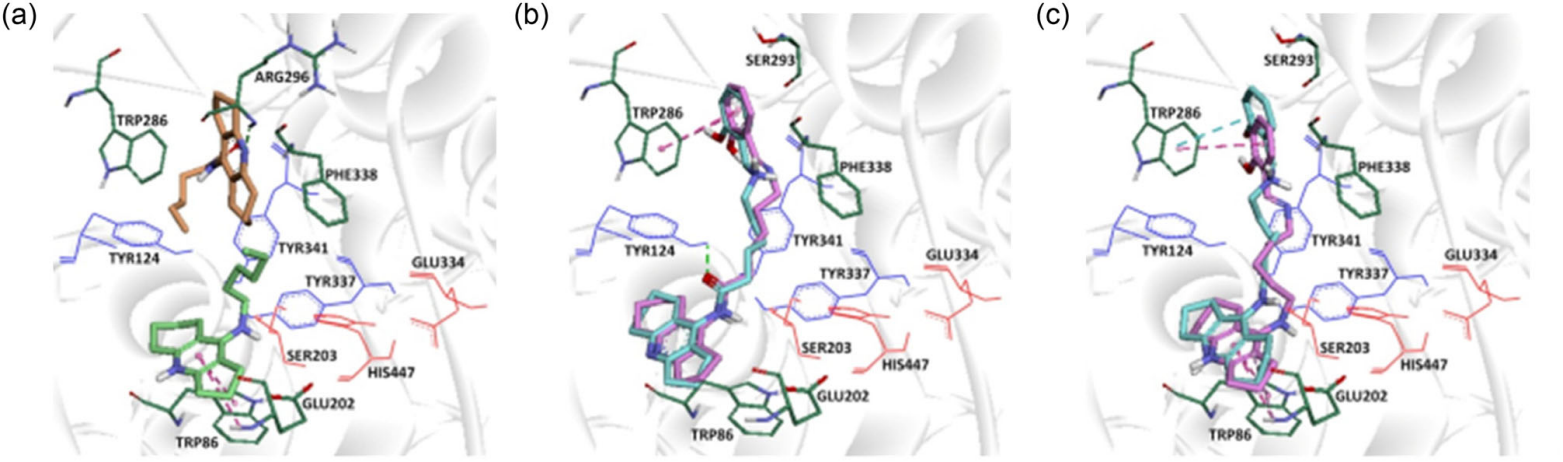
The most energetically favorable AChE binding poses of model compounds **3** (green) and **11** (brown) (a); of conjugates with an *N*‐acylalkylene spacer **9** (pink) and **10** (blue) (b); of conjugates with an *N*‐alkylene spacer **1** (pink) and **2** (blue) (c). Catalytic residues (Ser203, Glu334, His447) are shown as red lines, and the residues that constrict the PAS and form the “bottleneck” (Tyr124, Tyr337, Tyr341) are shown as blue lines.

To reveal the influence of the *N*‐acyl group on the binding mode of amiridine derivatives to the AChE, molecular docking for two model compounds **3** and **11** was performed (see Figure 3a). Molecular modeling showed that compound **11** with an *N*‐acylalkylene fragment and neutral amiridine pharmacophore was bound in the PAS due to the hydrogen bond (HB) between endocyclic *N*‐atom of the amiridine pharmacophore and protonated *N*‐atom of Arg296 main chain. Compound **3** with an *N*‐alkylene fragment and a protonated amiridine pharmacophore was bound in the CAS forming π–π stacking interaction with the aromatic side chain of Trp86. The docking pose of compound **3** was more energetically favorable than that of compound **11** because of the addition of an ionic interaction with the negatively charged side chain of Glu202.

The preferred docking poses of conjugates with an *N*‐acylalkylene spacer **9** and **10** were found to be similar, where the amiridine pharmacophore was located in the CAS and the second pharmacophore was bound in the PAS (see Figure 3b). These poses were stabilized due to a π–π stacking interaction of the second pharmacophore with the aromatic side chain of Trp286, whereas the amiridine pharmacophore did not form any specific interactions with CAS residues.

Conjugates **1** and **2** with an *N*‐alkylene spacer exhibited preferred docking poses that were similar to those of conjugates **9** and **10** with an *N*‐acylalkylene spacer, where the amiridine pharmacophore was bound in the CAS and the second pharmacophore was bound in the PAS (see Figure 3c). However, the docking poses of **1** and **2** were additionally stabilized by notable interactions of the protonated amiridine pharmacophore with CAS residues Trp86 and Glu202. Namely, it formed a π–π stacking interaction with the aromatic side chain of Trp86 and an ionic interaction with the negatively charged side chain of Glu202.

Thus, according to the molecular docking results for AChE, the protonated amiridine pharmacophore forms specific interactions with the AChE CAS residues (π–π stacking and ionic interactions) that are lacking in the case of the neutral amiridine pharmacophore. This furnishes an explanation for the experimental observations showing considerably higher anti‐AChE potency of conjugates with an *N*‐alkylene spacer compared to conjugates with an *N*‐acylalkylene linkage.

**FIGURE 4:**
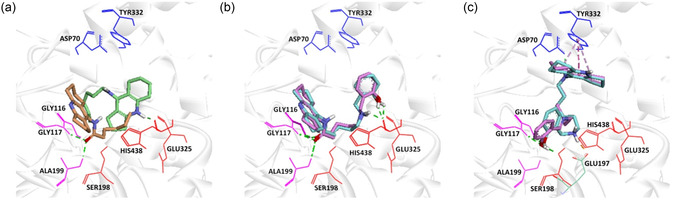
The most energetically favorable BChE binding poses of model compounds **3** (green) and **11** (brown) (a); conjugates with an *N*‐acylalkylene spacer **9** (pink) and **10** (blue) (b); conjugates with an *N*‐alkylene spacer **1** (pink) and **2** (blue) (c). Catalytic residues (Ser198, Glu325, His438) are shown as red lines, residues constricting the PAS at the entrance to the gorge (Asp70, Tyr332) are shown as blue lines, residues of the oxyanion hole (Gly116, Gly117, Ala199) are shown as pink lines.

Molecular docking showed that the model compound **11** with the *N*‐acyl group was effectively bound in BChE due to three hydrogen bonds between the carbonyl *O*‐atom of **11** and the protonated *N*‐atoms of the main chains of three residues of the oxyanion hole Gly116, Gly117, and Ala199 (see Figure 4a). This binding mode in the oxyanion hole is characteristic of the carbonyl oxygen. As a result, the decrease in the inhibitory activity of compounds with an *N*‐acyl group in BChE was less pronounced than in AChE.

The model compound **3** was bound in the CAS due to a hydrogen bond between the protonated *N*‐atom of the amiridine pharmacophore and an ionic interaction with the negatively charged Glu325 (see Figure 4a).

The most energetically favorable binding poses of the conjugates **9** and **10** with the *N*‐acylalkylene spacer are shown in Figure 4b. Both pharmacophores of the conjugates were bound in the CAS. The amiridine pharmacophore was bound in the oxyanion hole as in the case of the model compound **11**, and the second pharmacophore formed hydrogen bonds with His438 of the catalytic triad. These docking results furnished an explanation of the experimental observation: that is, binding of the amiridine fragment of the compounds with the *N*‐acylalkylene spacer in the oxyanion hole determines the relatively high anti‐BChE activity of these compounds.

The most energetically favorable binding poses of the conjugates **1** and **2** with an *N*‐alkylene spacer are shown in Figure 4c. The amiridine pharmacophore of both conjugates was bound in the PAS, forming π–π stacking interactions with the aromatic moiety of the side chain of Tyr332 and an ionic interaction with the negatively charged side chain of Asp70. The second pharmacophores were bound in the CAS due to HBs with Gly116, Gly117, and Ser198 residues. The protonated second pharmacophore of **2** formed additional ionic interaction with Glu197, which provides an explanation for the experimentally observed high anti‐BChE activity of compound **2**.

According to our molecular docking results, conjugates **1, 2** with an *N*‐alkylene spacer were bound both in the CAS and PAS of BChE (Figure 4c), a result that agrees with mixed‐type inhibition as found in the kinetics experiments [30]. At the same time, molecular docking results for conjugates **9, 10** with an *N*‐acyl spacer showed that these ligands were bound only in the CAS of BChE (Figure 4b). Although binding only to the CAS might have led to the expectation of a competitive interaction with the enzyme, we found that compounds **9** and **10** exhibited mixed‐type kinetics. However, this apparent paradox can be explained by examining the structure of the enzyme, as detailed below.

When BChE inhibitors are competitive in docking but show mixed‐type kinetics, it means they physically bind into the enzyme's catalytic active site (like a competitive inhibitor) but do not fully block the substrate from entering, thereby causing changes in both the enzyme's binding affinity *K* *_m_* and maximum reaction velocity *V*_max_. Both BChE and AChE have a tunnel (often referred to as a gorge) that connects the CAS to the PAS in each enzyme. However, the diameter of the gorge in BChE is significantly larger than that of AChE [41]. Consequently, some inhibitors of BChE do not strictly block the substrate from squeezing past them. Instead, such inhibitors bind to both the free enzyme and the enzyme–substrate complex, which is a characteristic of mixed‐type inhibition. Such dualistic behavior has been amply demonstrated for a variety of BChE inhibitors that position themselves in the CAS in docking simulations (mimicking a competitive profile), while kinetic assays reveal a mixed‐type of inhibition [42, 43, 44].

#### 2.2.7 Molecular Docking to Aβ_42_

Molecular docking was performed for three conformers (conformers 1, 2, and 3) of the monomeric *α*‐helical NMR solution structure of Aβ_42_ (PDB ID 1IYT) [45, 46]. It was found that molecular docking results were similar for all three conformers.

Our rationale for studying Aβ_42_ and selecting the monomeric structure PDB ID 1IYT as the target for our molecular docking studies of this peptide was based on the longstanding premise that Aβ_42_ is thought to be one of the key drivers of molecular pathology in AD [47, 48]. Furthermore, extensive evidence supports the idea that the early stages of Aβ_42_ aggregation (e.g., oligomers and protofibrils) play a pivotal role in the development of neurotoxicity [49, 50, 51]. Thus, structural studies and the search for inhibitors at these stages are particularly relevant. Therefore, we elected to study the monomeric form of the Aβ_42_ peptide at these initial stages of the pathological cascade, preceding the formation of insoluble fibrils [52, 53, 54].

Aβ_42_ is intrinsically disordered, and there are no experimentally derived structures of the monomer that have been obtained under physiological conditions [55].To address this limitation, we recently used an AlphaFold3 reproduction code to predict the structure of the peptide for use as a docking target for a series of ferrocene derivatives as potential anti‐AD therapeutic agents [56] However, there are three NMR structures available in the Protein Data Bank (1IYT, 1Z0Q, and 6SZF). Although these structures were obtained under seemingly nonphysiological conditions in aqueous solutions with varying amounts of the highly polar solvent, hexafluoroisopropanol (HFIP), the 1IYT structure that we chose to use for the present investigation has been successfully and repeatedly used as a target for molecular docking and virtual screening of potential inhibitors of Aβ_42_ self‐aggregation [52, 53, 54]**.** Moreover, in the present study, our molecular docking results obtained by using 1IYT as the peptide target were in agreement with our experimentally observed results on the inhibition of Aβ_42_ self‐aggregation.

**FIGURE 5:**
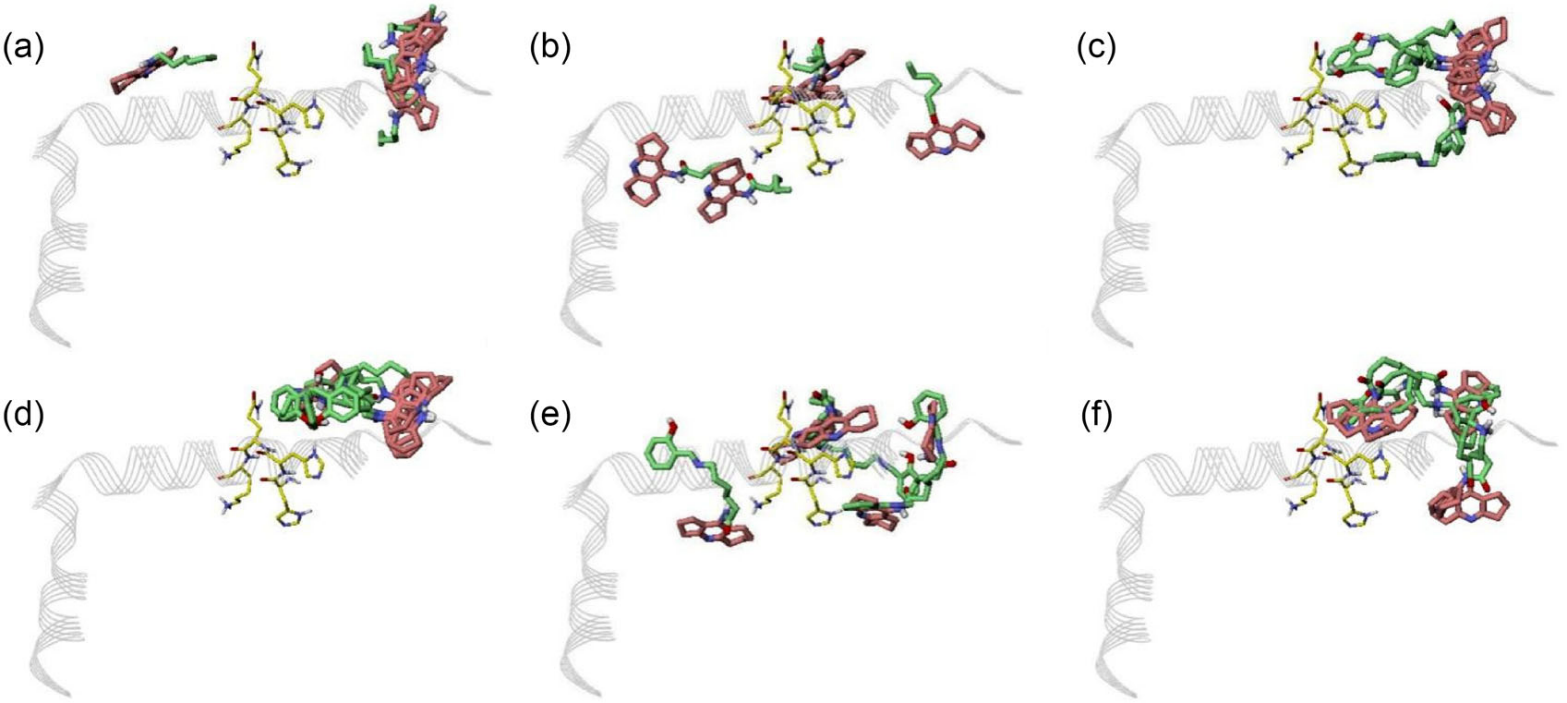
Five most energetically favorable Aβ_42_ (conformer 1) binding poses of model compounds **3** (a) and **11** (b); conjugates with *N*‐alkylene spacer **1** (c) and **2** (d); conjugates with *N*‐acylalkylene spacer **9** (e) and **10** (f). Carbon atoms of amiridine pharmacophore are shown in brown, other carbon atoms of the ligands are shown in green. Carbon atoms of the HHQK region of the Aβ_42_ peptide are shown in yellow.

Model compounds **3** and **11** demonstrated different binding trends to the Aβ_42_ peptide. Protonated **3** was preferentially bound to the negatively charged N‐terminus region (see Figure 5a), whereas neutral **11** did not exhibit a preferred binding region (see Figure 5b).

Similarly, conjugates with *N*‐acylalkylene and *N*‐alkylene spacers also showed different binding trends. Conjugates **1** and **2** with an *N*‐alkylene spacer demonstrated similar docking poses (see Figure 5c,d), where the protonated amiridine pharmacophore was bound to the N‐terminus region. In contrast, the second pharmacophore is anchored at the HHQK region [57], which is known to play a critical role in self‐aggregation [58, 59]. This binding mode effectively shielded the targeted HHQK region and enhanced the anti‐aggregation efficacy of conjugates with an *N*‐alkylene spacer. Protonation of the second pharmacophore in compound **2** resulted in a more energetically favorable pose than that of compound **1**, which corresponded to the higher anti‐aggregation efficacy of **2** that was observed experimentally.

Conjugates **9, 10** with an *N*‐acylalkylene spacer did not show any specific binding mode with shielding of the HHQK region (see Figure 5e,f), and these compounds did not exhibit any anti‐aggregation activity in the experimental determinations.

#### 2.2.8 Prediction of ADMET, Physicochemical, and PAINS Profiles

The results of our computational estimates of selected ADMET and physicochemical properties for compounds **1**, **2**, **9**, and **10** are shown in Table 2. Overall, the amide derivatives **9** and **10** are very similar to the corresponding noncarbonyl compounds **1** and **2**. The compounds had high predicted values for intestinal absorption, enabling their oral administration. For most of them, we could expect reasonable CNS activity in view of moderate or good predicted blood–brain barrier permeability (brain concentration is about 5%–95% of the plasma concentration).

**TABLE 2:** Predicted ADMET and physicochemical profiles of compounds **1, 2, 9**, and **10**.

	MW	LogP_ow_	pS_aq_	LogBB	HIA, %	hERG *pK*_*i*_	hERG *pIC*_50_	QED	PAINS
**1** a	391.56	5.42	5.63	−0.55	79	4.19	5.54	0.45	—
**2** a	393.57	4.58	4.51	−0.02	81	4.47	5.42	0.50	mannich_A(296)
**9**	405.54	4.20	5.26	−1.28	75	4.40	4.96	0.49	—
**10**	407.56	3.28	4.12	−0.76	77	4.65	4.89	0.54	mannich_A(296)
Amiridine	188.27	2.62	1.75	−0.58	92	4.34	4.44	0.68	—

Abbreviations: hERG *pIC*_50_, hERG potassium channel inhibitory activity [−log(M)]; hERG *pKi,* hERG potassium channel affinity [−log(M)]; HIA, human intestinal absorption [%]; LogBB, blood–brain barrier distribution; LogP_ow_, octanol‐water partition coefficient; MW, molecular weight; pS_aq_, aqueous solubility [−log(M)]; QED, quantitative estimate of drug‐likeness.

a Data for compounds **1, 2** are taken from the work [30].

The most notable distinction of amides is substantially lower predicted blood–brain barrier permeability. The cardiac toxicity risk parameters (hERG p*K*_*i*_ and pIC_50_) fell within 3.9–6.0 log units for all the analyzed compounds, which was within the lower and medium parts of their possible range (3–9 log units).

According to the commonly accepted drug‐likeness guidelines, the predicted lipophilicities and aqueous solubilities, as well as the molecular weights of the compounds, were within or close to the desirable range for potential drug compounds, although the LogP values in some cases violated the original Rule‐of‐5 limits (however, given that some of the compounds were outside of the model applicability domain, the predicted values were not fully reliable). The integral quantitative estimates of drug‐likeness (QED) were in the 0.4–0.6 range. The PAINS filter check revealed the “mannich_A(296)” alert for compounds **2** and **10**.

Consequently, the predicted ADMET, physicochemical, and PAINS properties of the compounds were acceptable for potential lead compounds in the discovery phase. Nevertheless, additional studies and structure optimization would be desirable to help maximize safety and improve the pharmacokinetic profile.

## 3 Conclusion

To gain mechanistic insight into the nature of the influence of *N*‐functionalization of amiridine on the biological properties of its conjugates, we synthesized conjugates of amiridine and salicylimine/amine **9, 10** with linkers containing an *N*‐acyl group at the amiridine fragment as analogs of the previously obtained conjugates **1, 2** with an *N*‐aminohexamethylene spacer.

In contrast to conjugates **1, 2**, which were effective inhibitors of AChE and BСhE, conjugates **9, 10** with an *N*‐acylalkylene spacer showed a substantial decrease in anti‐BChE activity (35‐fold for the imine and 8‐fold for the amine) and a more pronounced loss of anti‐AChE activity (inhibition 5.1% and 27.7% at 20 µM), as well as the disappearance of the ability to block Aβ_42_ self‐aggregation. Similar effects were observed for the model compounds *N*‐hexylamiridine **3** and its *N*‐acyl analog **11.**

However, *N*‐acyl‐functionalization did not diminish propidium displacement form the AChE PAS and only slightly lowered their antiradical activity, maintaining the higher activity of amines compared to imines.

The use of QM calculations and molecular docking furnished explanations for the experimentally observed effects. QM calculations showed that *N*‐acylation of amiridine led to a considerable decrease in proton affinity of its endocyclic *N*‐atom, and the amiridine pharmacophore in compounds **9**–**11** was predicted to be virtually unprotonated. According to results of molecular docking, this lack of protonation corresponded to a marked decrease in their anticholinesterase activity, especially anti‐AChE, as well as loss of the ability to block Aβ_42_ self‐aggregation.

A plausible explanation of the less pronounced reduction of anti‐BChE activity for *N*‐acyl conjugates **9, 10** and model compound **11** compared to anti‐AChE efficacy is the interaction of the carbonyl moiety of the conjugates with the BChE oxyanion hole as observed in the molecular docking results.

Molecular docking also furnished an explanation for the preservation of propidium displacement capacity from the *Ee*AChE PAS for *N*‐acyl conjugates **9, 10** by their binding of the salicylimine/amine fragment in the PAS site.

Radical‐scavenging activity of conjugates was less affected by the type of functionalization of the amiridine pharmacophore and depended to a greater extent on the presence of an imine or amine fragment in the molecule.

Overall, the results of the present study support our hypothesis that using an aminoalkylene linker is the more conducive strategy for the development of amiridine‐based conjugates as multitarget anti‐AD agents, while the use of *N*‐acyl functionalization may be an appropriate approach for obtaining more selective BChE inhibitors with moderate potency.

## 4 Experimental Section

All experiments were conducted in accordance with the standard protocols approved by IPAС of the Russian Academy of Sciences (RAS).

See the Supporting Information for the synthesis and characterization methods of new compounds, biological assay protocols, and molecular modeling method. The authors have cited additional references within the Supporting Information [60, 61, 62, 63, 64, 65, 66, 67, 68, 69, 70, 71, 72, 73, 74, 75, 76, 77, 78, 79, 80, 81, 82, 83, 84, 85, 86, 87, 88].

## Funding

This work was supported by the Russian Science Foundation (Project No. 24‐63‐00016, https://rscf.ru/project/24‐63‐00016/).

## Conflicts of Interest

The authors declare no conflicts of interest.

## Supporting information

Supplementary Material

## Data Availability

Data are available in the main article and the Supporting Information.
