# Supplementary material for: Influence of N‐Functionalization of Amiridine on the Biological Activity of Its Conjugates as Multitarget Agents for Potential Treatment of Alzheimer's Disease
Source: ChemMedChem. 2026 Jul 24;21(14):e70392. doi: 10.1002/cmdc.70392 (PMC13400319; doi:10.1002/cmdc.70392)

Influence of *N*-functionalization of amiridine on the biological activity of its conjugates as multitarget agents for potential treatment of Alzheimer's disease

Galina F. Makhaeva, Tatiana Y. Astakhova, Maria V. Grishchenko, Tatiana S. Shteinberg, Nadezhda V. Kovaleva, Natalia P. Boltneva, Elena V. Rudakova, Pavel G. Pronkin, Elena N. Timokhina, Eugene V. Radchenko, Evgeny V. Shchegolkov, Yanina V. Burgart, Victor I. Saloutin, Valery N. Charushin, Rudy J. Richardson

Table of Contents

1 Chemical part 3

2 Biological assays 6

In Vitro AChE, BChE, and CES inhibition 6

Kinetic study of BChE inhibition. Determination of steady-state inhibition constants 6

Propidium displacement studies 7

Inhibition of β-amyloid (1–42) ($A\beta_{42}$) self-aggregation 7

ABTS assay 7

FRAP assay 8

3 Molecular modeling studies 8

4 Prediction of ADMET, physicochemical, and PAINS profiles 9

References 9

Figure S1. NMR ^{1}H spectrum of 6-(1,3-dioxoisoindolin-2-yl)-*N*-(2,3,5,6,7,8-hexahydro-1*H*-cyclopenta[*b*]quinolin-9-yl)hexanamide 12

Figure S2. NMR ^{13}C spectrum of 6-(1,3-dioxoisoindolin-2-yl)-*N*-(2,3,5,6,7,8-hexahydro-1*H*-cyclopenta[*b*]quinolin-9-yl)hexanamide 13

Figure S3. IR spectrum of 6-(1,3-dioxoisoindolin-2-yl)-*N*-(2,3,5,6,7,8-hexahydro-1*H*-cyclopenta[*b*]quinolin-9-yl)hexanamide 14

Figure S4. NMR ^{1}H spectrum of 6-amino-*N*-(2,3,5,6,7,8-hexahydro-1*H*-cyclopenta[*b*]quinolin-9-yl)hexanamide 15

Figure S5. NMR ^{13}C spectrum of 6-amino-*N*-(2,3,5,6,7,8-hexahydro-1*H*-cyclopenta[*b*]quinolin-9-yl)hexanamide 16

Figure S6. IR spectrum of 6-amino-*N*-(2,3,5,6,7,8-hexahydro-1*H*-cyclopenta[*b*]quinolin-9-yl)hexanamide 17

Figure S7. NMR 1H spectrum of N-(2,3,5,6,7,8-hexahydro-1H-cyclopenta[b]quinolin-9-yl)-6-((2-hydroxybenzylidene)amino)hexanamide 18

Figure S8. NMR ^{13}C spectrum of *N*-(2,3,5,6,7,8-hexahydro-1*H*-cyclopenta[*b*]quinolin-9-yl)-6-((2-hydroxybenzylidene)amino)hexanamide 19

Figure S9. IR spectrum of *N*-(2,3,5,6,7,8-hexahydro-1*H*-cyclopenta[*b*]quinolin-9-yl)-6-((2-hydroxybenzylidene)amino)hexanamide 20

Figure S10. NMR ^{1}H spectrum of *N*-(2,3,5,6,7,8-hexahydro-1*H*-cyclopenta[*b*]quinolin-9-yl)-6-((2-hydroxybenzyl)amino)hexanamide 21

Figure S11. NMR ^{13}C spectrum of *N*-(2,3,5,6,7,8-hexahydro-1*H*-cyclopenta[b]quinolin-9-yl)-6-((2-hydroxybenzyl)amino)hexanamide 22

Figure S12. IR spectrum of *N*-(2,3,5,6,7,8-hexahydro-1*H*-cyclopenta[*b*]quinolin-9-yl)-6-((2-hydroxybenzyl)amino)hexanamide 23

Figure S13. NMR ^{1}H spectrum of *N*-(2,3,5,6,7,8-hexahydro-1*H*-cyclopenta[*b*]quinolin-9-yl)hexanamide 24

Figure S14. NMR ^{13}C spectrum of *N*-(2,3,5,6,7,8-hexahydro-1*H*-cyclopenta[*b*]quinolin-9-yl)hexanamide 25

Figure S15. IR spectrum of *N*-(2,3,5,6,7,8-hexahydro-1*H*-cyclopenta[*b*]quinolin-9-yl)hexanamide 26

Figure S16. HRMS spectrum of 6-(1,3-dioxoisoindolin-2-yl)-*N*-(2,3,5,6,7,8-hexahydro-1*H*-cyclopenta[*b*]quinolin-9-yl)hexanamide 27

Figure S17. HRMS spectrum of *N*-(2,3,5,6,7,8-hexahydro-1*H*-cyclopenta[*b*]quinolin-9-yl)hexanamide 27

1 Chemical part

Melting points were determined in open capillaries on a Stuart SMP30 melting point apparatus (Bibby Scientific Limited, Staffordshire, UK) and were uncorrected. The IR spectra were recorded on a Perkin Elmer Spectrum Two instrument (PerkinElmer, Waltham, MA, USA) using a frustrated total internal reflection accessory with a diamond crystal. The ^{1}H and ^{13}C NMR spectra were registered on a Bruker Avance^{III} 500 spectrometer (500 or 125 MHz, respectively) (Bruker). The internal standard was $SiMe_4$. The microanalyses (C, H, N) were carried out on a PerkinElmer PE 2400 series II elemental analyzer (PerkinElmer, Waltham, MA, USA). The high-resolution mass spectrometry (HRMS) was performed using a Bruker Daltonics MaXis Impact HD (Bruker, Karlsruhe, Germany) quadrupole time-of-flight mass spectrometer with negative electrospray ionization (ESI) from MeCN solutions, flow rate 0.35 ml·min^{-1} with parameters optimized for small molecules detection based on a pre-installed method for infusion analysis. The column chromatography was performed on silica gel 60 (0.062–0.2 mm) (Macherey-Nagel GmbH & Co KG, Duren, Germany).

Ethanol, chloroform, methylene chloride, toluene, sodium bicarbonate, hydrochloric acid, sodium hydroxide, sodium sulfate, sodium borohydride, ammonium hydroxide, and hydrazine hydrate were obtained from VEKTON AO (St. Petersburg, Russia). Hexane and acetonitrile were obtained from EKOS-1 (Moscow, Russia). Salicylic aldehyde was purchased from Alfa Aesar via Thermo Fisher Scientific (Kandel, Germany). The deuterated solvent $CDCl_3$ was acquired from SOLVEX LLC (Skolkovo Innovation Center, Moscow, Russia). All solvents, chemicals, and reagents were used without purification.

Amiridine **4** [1], 6-(1,3-dioxoisoindolin-2-yl)hexanoyl chloride **5** [2], hexanoic acid chloride [3] were synthesized by referring to the previously published method.

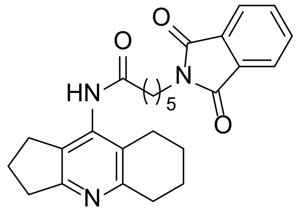

Synthesis of 6-(1,3-dioxoisoindolin-2-yl)-*N*-(2,3,5,6,7,8-hexahydro-1*H*-cyclopenta[*b*]quinolin-9-yl)hexanamide (6).

A mixture of amiridine **4** (1.00 g, 5.3 mmol) and 6-(1,3-dioxoisoindolin-2-yl)hexanoyl chloride **5** (3.00 g, 10.7 mmol) was stirred at 95 °C for 10 h. $CHCl_3$ (100 mL) was added to the reaction mixture after cooling. Then, 10% (w/v) aqueous $NaHCO_3$ solution (75 mL) was added with stirring and ice-cooling. The organic layer was separated and washed with 10% (w/v) aqueous $NaHCO_3$ solution (2 × 75 mL), H_2O (3 × 75 mL), dried over anhydrous Na_2SO_4, and evaporated. The precipitate was washed with MeCN (3 × 20 mL), MeCN/EtOH mixture (20:1, 21 mL) and dried. Yield: 1.85 g (81%), white solid, Mp: 221–226 °C (with decomp.), R_f 0.73 ($CHCl_3$–EtOH–NH_4OH, 25:1:0.1). ^{1}H NMR (500 MHz, $CDCl_3$) δ: 1.42–1.48 (m, 2H, CH_2), 1.73–1.86 (m, 8H, $4CH_2$), 2.02 (quintet, 2H, *J* 7.5 Hz, CH_2),

2.39–2.42 (m, 2H, CH_2), 2.56–2.58 (m, 2H, CH_2), 2.74 (t, 2H, *J* 7.4 Hz, CH_2), 2.88–2.91 (m, 2H, CH_2), 2.95 (t, 2H, *J* 7.6 Hz, CH_2), 3.72 (t, 2H, *J* 7.1 Hz, C(O)CH_2), 6.86 (br. s, 1H, NH), 7.69–7.80 (m, 4H, CH_{Ar}). ^{13}C NMR (125 MHz, $CDCl_3$) δ: 22.6, 22.8, 22.9, 24.5, 25.1, 26.1, 28.0, 29.9, 32.7, 34.4, 36.5, 37.5, 123.1, 123.5, 130.1, 132.0, 133.9, 139.1, 156.1, 163.8, 168.5 (C=O), 170.2 (C=O). IR (*v*/cm^{-1}): 3257, 2941, 2860 (NH, CH), 1767, 1704 (C=O), 1652, 1569, 1513, 1429, 1395 (NH, C=C, C=N) cm^{-1}. HRMS (NEG): m/z $[M–H]^-$ calcd 430.2136, found 430.2136. Found (%): C, 72.15; H, 6.78; N, 9.56. Calc. for $C_{26}H_{29}N_3O_3$ (%): C, 72.37; H, 6.77; N, 9.74.

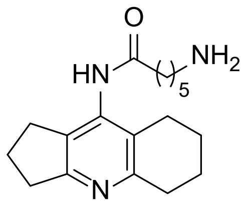

Synthesis of 6-amino-*N*-(2,3,5,6,7,8-hexahydro-1*H*-cyclopenta[*b*]quinolin-9-yl)hexanamide (7). A mixture of compound **6** (1.00 g, 2.32 mmol) and hydrazine hydrate (0.20 mL, 4.20 mmol) in EtOH was refluxed for 2 h. The precipitate that formed after cooling the reaction mixture was filtered off and dried. 2 N HCl (30 mL) was added to the precipitate, solid NaOH was added to pH 9, the mixture was extracted with $CHCl_3$ (3 × 50 mL), dried over anhydrous Na_2SO_4, and evaporated. Yield: 0.522 g (75%), white solid, Mp: 151–153 °C. ^{1}H NMR (500 MHz, DMSO-d_6) δ: 1.31 (m, 4H, 2CH_2), 1.55–1.61 (m, 2H, CH_2), 1.66–1.77 (m, 4H, 2CH_2), 1.96 (quintet, 2H, *J* 7.5 Hz, CH_2), 2.31 (t, 2H, *J* 7.3 Hz, CH_2), 2.53–2.55 (m, 4H, 2CH_2), 2.66 (t, 2H, *J* 7.4 Hz, CH_2), 2.74–2.77 (m, 2H, CH_2), 2.82 (t, 2H, *J* 7.6 Hz, CH_2), 9.38 (s, 1H, NH) (the signals of the protons of NH_2- group were not observed due to deuterium exchange with the solvent). ^{13}C NMR (125 MHz, DMSO-d_6) δ: 22.2, 22.4, 22.5, 23.80, 25.3, 26.0, 29.1, 32.3, 33.0, 33.7, 35.5, 41.5, 124.1, 130.0, 140.1, 155.1, 162.4, 170.3 (C=O). IR (*v*/cm^{-1}): 3255, 2927, 2854 (NH, CH), 1659 (C=O), 1602, 1571, 1519, 1438, 1397 (NH, C=C, C=N) cm^{-1}. Found (%): C, 71.54; H, 9.15; N, 13.75. Calc. for $C_{26}H_{29}N_3O_3$ (%): C, 71.72; H, 9.03; N, 13.94.

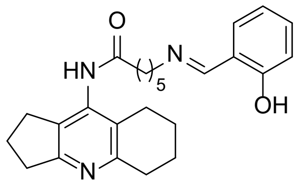

Synthesis *N*-(2,3,5,6,7,8-hexahydro-1*H*-cyclopenta[*b*]quinolin-9-yl)-6-((2-hydroxybenzylidene)amino)hexanamide (9). A solution of salicylic aldehyde **8** (0.20 mL, 2.0 mmol) in anhydrous toluene (75 mL) and a solution of compound **7** (0.602 g, 2.0 mmol) in anhydrous EtOH (2 mL) were refluxed with azeotropic distillation of water for 6 h using a Dean-Stark trap, then toluene was distilled off. Hexane was added to the residue, and the resulting precipitate was filtered off and dried. The product was isolated by column chromatography (eluent – CH_2Cl_2/EtOH (40:1)). Yield: 0. 356 g (44%), pale yellow solid, Mp: 155–157 °C, R_f 0.68 ($CHCl_3$–EtOH–NH_4OH, 5:1:0.1). ^{1}H NMR (500 MHz, $CDCl_3$) δ: 1.48–1.54 (2H, м, CH_2); 1.68 (1H, br. s, NH); 1.74–1.86 (m, 8H, 2CH_2), 2.04 (quintet, 2H, *J* 7.5 Hz, CH_2), 2.39–2.42 (m, 2H, CH_2), 2.54–2.56 (m, 2H, CH_2), 2.76 (t, 2H, *J* 7.4 Hz, CH_2), 2.88–2.90 (m, 2H, CH_2), 2.96 (t, 2H, *J* 7.6 Hz, CH_2), 3.62 (t, 2H, *J* 6.5 Hz, CH_2), 6.86 (unsolv. td, 1H, *J* 7.5, 0.9 Hz,

CH_{Ar}), 6.92 (d, 1H, *J* 8.2 Hz, CH_{Ar}), 7.23 (dd, 1H, *J* 7.6, 1.6 Hz, CH_{Ar}), 7.26 (td, 1H, *J* 7.6, 1.7 Hz, CH_{Ar}), 8.35 (s, 1H, N=CH), 13.65 (br. s, 1H, OH). ^{13}C NMR (125 MHz, $CDCl_3$) δ: 22.6, 22.8, 22.9, 24.5, 25.4, 26.8, 29.9, 30.4, 32.7, 34.5, 36.7, 59.2, 117.0, 118.5, 118.7, 123.6, 130.1, 131.1, 132.2, 139.1, 156.2, 161.3, 163.9, 164.8, 170.4 (C=O). IR (v/cm^{-1}): 3254, 2936, 2853 (NH, CH, OH), 1656 (C=O), 1636, 1568, 1517, 1463, 1396 (NH, C=C, C=N) cm^{-1}. Found (%): C, 73.95; H, 7.98; N, 10.29. Calc. for $C_{26}H_{29}N_3O_3$ (%): C, 74.04; H, 7.71; N, 10.36.

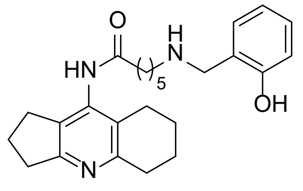

Synthesis of *N*-(2,3,5,6,7,8-hexahydro-1*H*-cyclopenta[*b*]quinolin-9-yl)-6-((2-hydroxybenzyl)amino)hexanamide (10). $NaBH_4$ (0.029 g, 0.75 mmol) was added to a solution of compound **9** (0.202 g, 0.50 mmol) in EtOH (20 mL). The reaction mixture was stirred at room temperature for 2 h and evaporated. CH_2Cl_2 (25 mL) was added to the residue, the solution was filtered to remove $NaBH_4$, the filtrate was evaporated, and dried. Yield: 0. 0.175 g (86%), white solid, Mp: 57–59 °C, R_f 0.56 ($CHCl_3$–EtOH–NH_4OH, 25:1:0.1). ^{1}H NMR (500 MHz, $CDCl_3$) δ: 1.43–1.49 (m, 2H, CH_2), 1.57–1.63 (m, 2H, CH_2), 1.72–187 (m, 6H, 3CH_2), 2.07 (quintet, 2H, *J* 7.6 Hz, CH_2), 2.37–2.43 (m, 2H, CH_2), 2.57 (t, 2H, *J* 6.2 Hz, CH_2), 2.71 (t, 2H, *J* 6.9 Hz, CH_2), 2.78 (t, 2H, *J* 7.4 Hz, CH_2), 2.89–2.91 (m, 2H, CH_2), 2.98 (t, 2H, *J* 6.2 Hz, CH_2), 4.00 (s, 2H, NH–$C\underline{H_2}$), 6.76–6.80 (m, 2H, CH_{Ar}), 6.91 (br. s, 1H, OH), 6.82–6.99 (m, 1H, CH_{Ar}), 7.15 (td, 1H, *J* 8.0, 1.6 Hz, CH_{Ar}) (the signal of the proton of NH-group was not observed due to deuterium exchange with the solvent). ^{13}C NMR (125 MHz, $CDCl_3$) δ: 22.6, 22.8, 23.0, 24.5, 25.4, 26.6, 29.1, 29.9, 32.7, 34.5, 36.7, 48.3, 52.7, 116.3, 119.0, 122.4, 123.6, 128.3, 128.7, 130.1, 139.1, 156.2, 158.2, 163.9, 170.4 (C=O). IR (v/cm^{-1}): 3230, 2929, 2856 (NH, CH, OH), 1659 (C=O), 1592, 1572, 1514, 1456, 1399 (NH, C=C, C=N). Found (%): C, 73.40; H, 8.36; N, 10.07. Calc. for $C_{26}H_{29}N_3O_3$ (%): C, 73.68; H, 8.16; N, 10.31.

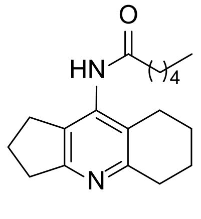

Synthesis *of* *N*-(2,3,5,6,7,8-hexahydro-1*H*-cyclopenta[*b*]quinolin-9-yl)hexanamide (11). A mixture of hexanoic acid chloride (1 ml, 7 mmol) and amiridine **4** (0.250 g, 1.3 mmol) was stirred at 95 °C for 8 h. After cooling, $CHCl_3$ (30 ml) was added to the reaction mixture. Then, 10% (w/v) aqueous $NaHCO_3$ solution (20 ml) was added with stirring and ice-cooling. The organic layer was separated and washed with 10% (w/v) aqueous $NaHCO_3$ solution (2 × 20 ml), water (3 × 20 ml), dried over anhydrous Na_2SO_4, and evaporated. The residue was purified by column chromatography (eluent $CHCl_3$/EtOH/NH_4OH (100:1:0.1, then 50:1:0.1)). The product was washed with hexane and dried. Yield: 0.245 g (66%), white solid, Mp: 175–176 °C, R_f 0.87 ($CHCl_3$–EtOH–NH_4OH, 25:1:0.1). ^{1}H NMR (500 MHz, $CDCl_3$) δ: 0.91–0.94 (m, 3H, CH_3), 1.36–1.39 (m, 4H, 2CH_2), 1.71–1.87 (m, 8H, 3CH_2+H_2O), 2.08 (quintet, 2H, *J* 7.5 Hz, CH_2), 2.39 (m,

2H, CH_2), 2.56–2.58 (m, 2H, CH_2), 2.79 (t, 2H, *J* 7.7 Hz, CH_2), 2.89–2.91 (m, 2H, CH_2), 2.98 (t, 2H, *J* 7.6 Hz, CH_2), 6.81 (br. s, 1H, NH). ^{13}C NMR (125 MHz, $CDCl_3$) δ: 13.9, 22.4, 22.6, 22.8, 23.0, 24.5, 25.6, 29.9, 31.4, 32.7, 34.5, 36.9, 123.5, 130.1, 139.1, 156.2, 163.9, 170.6 (C=O). IR (ν/cm^{-1}): 3262, 2952, 2930, 2860 (NH, CH), 1661 (C=O), 1603, 1567, 1519, 1439, 1398 (NH, C=C, C=N). HRMS (NEG): m/z [M–H]$^-$ calcd 285.1972, found 285.1973. Found (%): C, 75.28; H, 9.19; N, 9.60. Calc. for $C_{26}H_{29}N_3O_3$ (%): C, 75.48; H, 9.15; N, 9.78.

2 Biological assays

In Vitro AChE, BChE, and CES inhibition

The following items were purchased from Sigma-Aldrich: porcine liver CES, acetylthiocholine iodide (ATCh), butyrylthiocholine iodide (BTCh), 5,5′-dithio-bis-(2-nitrobenzoic acid) (DTNB), 4-nitrophenyl acetate (4-NPA), and bis(4-nitrophenyl) phosphate (BNPP). Human erythrocyte AChE and equine serum BChE were purchased from NPK Melamed LLC (Russia). We measured the activity of AChE and BChE according to the colorimetric Ellman procedure [4] (λ = 412 nm), as described in detail in [5]. CES activity was assessed as described in [5] by following the release of 4-nitrophenol spectrophotometrically (λ = 405 nm) using 4-NPA as a substrate. Freshly prepared solutions of the enzymes were used, which retained a constant activity during the experiment (2–2.5 h). Chromophore absorbances were measured with a SPECTROStar Nano microplate reader (BMG Labtech). DMSO (2% v/v) was employed as the solvent; the concentration used did not alter the activities of the enzymes (data not shown). Initially, we used a single concentration of 20 μM for all compounds. Subsequently, IC_{50} values (μM) were determined for the most active compounds against AChE, BChE, and CES.

Kinetic study of BChE inhibition. Determination of steady-state inhibition constants

We assessed the mechanisms of BChE inhibition by performing a thorough analysis of enzyme kinetics [5]. After a 5 min incubation at 25°C (for temperature equilibration) with three increasing concentrations of inhibitor and six decreasing substrate concentrations, the residual enzyme activity was measured as described above for enzymatic assays. Linear regression of 1/V versus 1/[S] double reciprocal (Lineweaver–Burk) plots were used to determine the inhibition constants for the competitive component (K_i) and noncompetitive component (αK_i).

Propidium displacement studies

The ability of the test compounds to competitively displace propidium was evaluated by a fluorescence method [6], as described in detail in [7]. Propidium iodide, donepezil, and electric eel AChE (*Ee*AChE, type VI-S, lyophilized powder) were purchased from Sigma-Aldrich (Saint Louis, MO, USA). After 15 min of incubation of the test compounds at a concentration of 20 µM with a 7 µM solution of *Ee*AChE in 1 mM Tris-HCl buffer, pH 8.0, 25 °C, propidium iodide (final concentration 8 µM) was added. Then, the solutions were incubated for 15 min and the fluorescence spectrum was recorded (530 nm (excitation) and 600 nm (emission)). Donepezil was the reference compound. Measurements were performed in triplicate on a FLUOStar Optima microplate reader (BMG LabTech, Ortenberg, Germany)

Inhibition of β-amyloid (1–42) ($A\beta_{42}$) self-aggregation

Inhibition of $A\beta_{42}$ self-aggregation by test compounds was studied using the thioflavin T (ThT) fluorescence method [8-10] with minor modifications as described in detail in [11]. Lyophilized HFIP-pretreated $A\beta_{42}$ (GenicBio Limited (Shanghai, China), 1 mg) was dissolved in DMSO to obtain a stable 500 µM solution. The samples of 50 µM $A\beta_{42}$ in 215 mM Na-phosphate buffer pH 8.0 were incubated for 24 h at 37°C in the absence or presence of 100 µM test compounds. Myricetin in the same concentration was used as reference. After incubation, 5 µM ThT in 50 mM glycine-NaOH buffer pH 8.5 was added, and the fluorescence was measured at 440 nm (exc.) and 485 nm (emis.) with a FLUOStar Optima microplate reader (BMG LabTech, Ortenberg, Germany). The blanks consisted of 215 mM Na-phosphate buffer, pH 8.0, 20% (v/v) DMSO or test compounds, respectively. Each assay was run in triplicate. The degree of inhibition of $A\beta_{42}$ self-aggregation by the studied compounds (%) was calculated by the following formula:

$\%\ inhibition = 100 - (IF_i / IF_o) \times 100$,

where IF_i and IF_o are the fluorescence intensities obtained for $A\beta_{42}$ in the presence or absence of inhibitor, respectively, after subtracting the fluorescence of respective blanks.

ABTS assay

Radical scavenging activity of the compounds was assessed using the ABTS radical cation ($ABTS^{\bullet+}$) decolorization assay in accordance with the method of Re et al. [12] with minor modifications, as described in detail earlier [13].

ABTS (2,2'-azinobis-(3-ethylbenzothiazoline-6-sulfonic acid) diammonium salt) was purchased from Tokyo Chemical Industry Co. Ltd. (Tokyo, Japan). Potassium persulfate (di-

potassium peroxydisulfate), Trolox (6-hydroxy-2,5,7,8-tetramethychroman-2-carboxylic acid), ascorbic acid, DMSO and HPLC-grade ethanol were obtained from Sigma-Aldrich Chemical Co. (St. Louis, MO, USA). Aqueous solutions were prepared using deionized water. To prepare the solution of $ABTS^{•+}$, equal volumes of aqueous solutions of 7 mM ABTS and 2.45 mM potassium persulfate were incubated for 12–16 h at room temperature in the dark. Radical scavenging capacity of the compounds was determined by mixing 10 μL of compound solution in DMSO with 240 μL of $ABTS^{•+}$ working solution in ethanol (100 μM final concentration). The reduction in $ABTS^{•+}$ absorbance was measured spectrophotometrically at 734 nm using an xMark UV/VIS microplate spectrophotometer (Bio-Rad, Hercules, CA, USA) for 1 h with an interval of 10 min compared to a standard synthetic antioxidant Trolox (6-hydroxy-2,5,7,8-tetramethychroman-2-carboxylic acid). Ascorbic acid was used as a positive control. The antioxidant activity of the compounds was reported as Trolox equivalent antioxidant capacity (TEAC values) as the ratio of the slopes of the concentration−response curves, test compound/Trolox.

FRAP assay

The FRAP (Ferric Reducing Antioxidant Power) assay [14] was modified to be performed in 96-well microplates as described previously in detail [15]. A total of 10 μL (0.5 mM) of the tested compound or reference compound was mixed with 240 μL of the FRAP reagent, and the absorbance of the mixture was measured spectrophotometrically (λ = 593 nm) with a SPECTROStar Nano microplate reader (BMG LabTech), after a 1 h incubation at 37°C against a blank. Trolox was used as a reference compound. The antioxidant activity (AOA) was calculated and expressed as Trolox equivalents (TE) — the values calculated as the ratio of the concentrations of Trolox and the test compound resulting in the same effect on ferric-reducing activity.

All plots, linear regressions and values of IC_{50}, were determined using Origin 6.1 for Windows, OriginLab (Northampton, MA, USA). Results were calculated using GraphPad Prism version 6.05 for Windows, GraphPad Software (La Jolla, CA, USA) and presented as mean ± SEM.

3 Molecular modeling studies

Molecular docking was performed using the Autodock 4.2.6 tool [16]. The docking study was performed in 1000 runs to obtain the best statistics, with the AMBER force field, [17] using the Lamarckian genetic algorithm. [18] In the molecular docking experiment, the proteins and ligands were the rigid and flexible parts of the system, respectively.

For docking to AChE, the structure of recombinant human acetylcholinesterase in the apo state PDB ID = 4EY4 was used. [19] The cubic simulation cell was chosen to include the entire active site gorge, with the center of the cell coordinates at x = -14.428, y = -42.923, z = 27.213 Å and edge dimensions 22.5 x 22.5 x 22.5 Å.

For docking to BChE, the PDB structure of human butyrylcholinesterase 1P0I was used; [20] the coordinates of the center of the cuboid simulation cell were x = 136.936, y = 116.711, z = 43.726 Å, and the cell edge dimensions (x, y, z) were 15 x 20 x 18 Å.

The ligand structures were taken from quantum-chemistry calculations in the Orca 6.1.0 package [21]. The structures were optimized by the DFT method using the B3LYP functional [22-23] with Grimme correction D3BJ [24] and basis set def2-TZVP [25]. The minimum energy structures were confirmed by the absence of negative frequencies in the calculated phonon spectra. To estimate the protonation state of the studied compounds the proton affinity (PA) values were calculated. To improve the accuracy of the PA calculations, single-point Hartree-Fock calculations with the def2-QZVPP basis set were used [25]. Solvent effects were taken into account within the SMD model [26]. The geometry and Mulliken atomic charges obtained in quantum-chemistry calculations were used in molecular docking simulations.

4 Prediction of ADMET, physicochemical, and PAINS profiles

Lipophilicity ($LogP_{ow}$) and aqueous solubility (pS) were estimated by the ALogPS 3.0 neural network model implemented in the OCHEM platform [27]. Human intestinal absorption (HIA) [28], blood–brain barrier distribution/permeability (LogBB) [29] [30], and hERG-mediated cardiac toxicity risk (channel affinity pK_i and inhibitory activity pIC_{50}) [31] were estimated using the integrated online service for the prediction of ADMET properties [32]. This service implements predictive QSAR models based on accurate and representative training sets, fragmental descriptors, and artificial neural networks. The quantitative estimate of drug-likeness (QED) values [33] were calculated and the Pan Assay INterference compoundS (PAINS) alerts were checked using RDKit version 2021.09.2 software [34].

References

[1] V. P. Chernyshev, A. S. Kovaleva, N. V. Kasatkin, V. S. Ivanov, S. D. Karakotov, "Method of producing 9-amino-2,3,5,6,7,8-hexahydro-1h-cyclopent[*b*]quinoline" Patent RU2659389C1, **2018**, priority of 19.12.2017

[2] R. Wang, S. An, Y. X. Xin, Y. Y. Jiang, W. H. Liu, "Redox-Neutral Umpolung Synthesis of alpha-Functionalized Amides" *JACS Au* **2024**, *4*, 4435-4444. doi 10.1021/jacsau.4c00767.

[3] E. A. Kuznetsova, N. M. Sazonova, S. V. Nikitin, L. A. Zhmurenko, T. A. Gudasheva, "Synthesis of the potential dipeptide neuroleptic dilept and its active metabolite" *Pharm. Chem. J.* **2013**, *47*, 243-246. doi 10.1007/s11094-013-0937-y.

[4] G. L. Ellman, K. D. Courtney, V. Andres, Jr., R. M. Feather-Stone, "A new and rapid colorimetric determination of acetylcholinesterase activity" *Biochem. Pharmacol.* **1961**, *7*, 88-95. doi 10.1016/0006-2952(61)90145-9.
[5] G. F. Makhaeva, N. V. Kovaleva, N. P. Boltneva, S. V. Lushchekina, E. V. Rudakova, T. S. Stupina, A. A. Terentiev, I. V. Serkov, A. N. Proshin, E. V. Radchenko, V. A. Palyulin, S. O. Bachurin, R. J. Richardson, "Conjugates of tacrine and 1,2,4-thiadiazole derivatives as new potential multifunctional agents for Alzheimer's disease treatment: Synthesis, quantum-chemical characterization, molecular docking, and biological evaluation" *Bioorg. Chem.* **2020**, *94*, 103387. doi 10.1016/j.bioorg.2019.103387.
[6] P. Taylor, S. Lappi, "Interaction of fluorescence probes with acetylcholinesterase. Site and specificity of propidium binding" *Biochemistry* **1975**, *14*, 1989-1997. doi 10.1021/bi00680a029.
[7] G. F. Makhaeva, N. V. Kovaleva, N. P. Boltneva, S. V. Lushchekina, T. Y. Astakhova, E. V. Rudakova, A. N. Proshin, I. V. Serkov, E. V. Radchenko, V. A. Palyulin, S. O. Bachurin, R. J. Richardson, "New Hybrids of 4-Amino-2,3-polymethylene-quinoline and p-Tolylsulfonamide as Dual Inhibitors of Acetyl- and Butyrylcholinesterase and Potential Multifunctional Agents for Alzheimer's Disease Treatment" *Molecules* **2020**, *25*. doi 10.3390/molecules25173915.
[8] H. LeVine, 3rd, "Quantification of beta-sheet amyloid fibril structures with thioflavin T" *Meth. Enzymol.* **1999**, *309*, 274-284. doi 10.1016/s0076-6879(99)09020-5.
[9] P. Muñoz-Ruiz, L. Rubio, E. García-Palomero, I. Dorronsoro, M. del Monte-Millán, R. Valenzuela, P. Usán, C. de Austria, M. Bartolini, V. Andrisano, A. Bidon-Chanal, M. Orozco, F. J. Luque, M. Medina, A. Martínez, "Design, Synthesis, and Biological Evaluation of Dual Binding Site Acetylcholinesterase Inhibitors: New Disease-Modifying Agents for Alzheimer's Disease" *J. Med. Chem.* **2005**, *48*, 7223-7233. doi 10.1021/jm0503289.
[10] M. Biancalana, S. Koide, "Molecular mechanism of Thioflavin-T binding to amyloid fibrils" *Biochim. Biophys. Acta - Proteins Proteomics* **2010**, *1804*, 1405-1412. doi 10.1016/j.bbapap.2010.04.001.
[11] G. F. Makhaeva, N. V. Kovaleva, E. V. Rudakova, N. P. Boltneva, M. V. Grishchenko, S. V. Lushchekina, T. Y. Astakhova, O. G. Serebryakova, E. N. Timokhina, E. F. Zhilina, E. V. Shchegolkov, M. V. Ulitko, E. V. Radchenko, V. A. Palyulin, Y. V. Burgart, V. I. Saloutin, S. O. Bachurin, R. J. Richardson, "Conjugates of Tacrine and Salicylic Acid Derivatives as New Promising Multitarget Agents for Alzheimer's Disease" *Int. J. Mol. Sci.* **2023**, *24*. doi 10.3390/ijms24032285.
[12] R. Re, N. Pellegrini, A. Proteggente, A. Pannala, M. Yang, C. Rice-Evans, "Antioxidant activity applying an improved ABTS radical cation decolorization assay" *Free Radic. Biol. Med.* **1999**, *26*, 1231-1237. doi 10.1016/s0891-5849(98)00315-3.
[13] G. F. Makhaeva, N. A. Elkina, E. V. Shchegolkov, N. P. Boltneva, S. V. Lushchekina, O. G. Serebryakova, E. V. Rudakova, N. V. Kovaleva, E. V. Radchenko, V. A. Palyulin, Y. V. Burgart, V. I. Saloutin, S. O. Bachurin, R. J. Richardson, "Synthesis, molecular docking, and biological evaluation of 3-oxo-2-tolylhydrazinylidene-4,4,4-trifluorobutanoates bearing higher and natural alcohol moieties as new selective carboxylesterase inhibitors" *Bioorg. Chem.* **2019**, *91*, 103097. doi 10.1016/j.bioorg.2019.103097.
[14] I. F. Benzie, J. J. Strain, "Ferric reducing/antioxidant power assay: direct measure of total antioxidant activity of biological fluids and modified version for simultaneous measurement of total antioxidant power and ascorbic acid concentration" *Meth. Enzymol.* **1999**, *299*, 15-27. doi 10.1016/s0076-6879(99)99005-5.
[15] G. F. Makhaeva, N. V. Kovaleva, E. V. Rudakova, N. P. Boltneva, S. V. Lushchekina, Faingold, II, D. A. Poletaeva, Y. V. Soldatova, R. A. Kotelnikova, I. V. Serkov, A. K. Ustinov, A. N. Proshin, E. V. Radchenko, V. A. Palyulin, R. J. Richardson, "New Multifunctional Agents Based on Conjugates of 4-Amino-2,3-polymethylenequinoline and Butylated Hydroxytoluene for Alzheimer's Disease Treatment" *Molecules* **2020**, *25*, 5891. doi 10.3390/molecules25245891.
[16] G. M. Morris, R. Huey, W. Lindstrom, M. F. Sanner, R. K. Belew, D. S. Goodsell, A. J. Olson, "AutoDock4 and AutoDock Tools4: automated docking with selective receptor flexibility" *J. Comp. Chem.* **2009**, *30*, 2785–2791. doi 10.1002/jcc.21256.
[17] D. A. Case, H. M. Aktulga, K. Belfon, D. S. Cerutti, G. A. Cisneros, V. W. D. Cruzeiro, N. Forouzesh, T. J. Giese, A. W. Gotz, H. Gohlke, S. Izadi, K. Kasavajhala, M. C. Kaymak, E. King, T. Kurtzman, T. S. Lee, P. Li, J. Liu, T. Luchko, R. Luo, M. Manathunga, M. R. Machado, H. M. Nguyen, K. A. O'Hearn, A. V. Onufriev, F. Pan, S. Pantano, R. Qi, A. Rahnamoun, A. Risheh, S. Schott-Verdugo, A. Shajan, J. Swails, J. Wang, H. Wei, X. Wu, Y. Wu, S. Zhang, S. Zhao, Q. Zhu, T. E. Cheatham, 3rd, D. R. Roe, A. Roitberg, C. Simmerling, D. M. York, M. C. Nagan, K. M. Merz, Jr., "AmberTools" *J. Chem. Inf. Model.* **2023**, *63*, 6183-6191. doi 10.1021/acs.jcim.3c01153.
[18] B. J. Ross, in *Practical handbook of genetic algorithms*, CRC Press, **2019**, pp. 1-16.
[19] J. Cheung, M. J. Rudolph, F. Burshteyn, M. S. Cassidy, E. N. Gary, J. Love, M. C. Franklin, J. J. Height, "Structures of Human Acetylcholinesterase in Complex with Pharmacologically Important Ligands" *J. Med. Chem.* **2012**, *55*, 10282-10286. doi 10.1021/jm300871x.

[20] Y. Nicolet, O. Lockridge, P. Masson, J. C. Fontecilla-Camps, F. Nachon, "Crystal structure of human butyrylcholinesterase and of its complexes with substrate and products" *J. Biol. Chem.* **2003**, *278*, 41141-41147. doi 10.1074/jbc.M210241200.
[21] F. Neese, "Software update: The ORCA program system—Version 5.0" *WIREs Comput. Mol. Sci.* **2022**, *12*. doi 10.1002/wcms.1606.
[22] A. D. Becke, "Density-functional thermochemistry. III. The role of exact exchange" *J. Chem. Phys.* **1993**, *98*, 5648-5652. doi 10.1063/1.464913.
[23] C. Lee, W. Yang, R. G. Parr, "Development of the Colle-Salvetti correlation-energy formula into a functional of the electron density" *Phys. Rev. B Condens. Matter* **1988**, *37*, 785-789. doi 10.1103/physrevb.37.785.
[24] S. Grimme, J. Antony, S. Ehrlich, H. Krieg, "A consistent and accurate ab initio parametrization of density functional dispersion correction (DFT-D) for the 94 elements H-Pu" *J. Chem. Phys.* **2010**, *132*, 154104. doi 10.1063/1.3382344.
[25] A. Hellweg, C. Hättig, S. Höfener, W. Klopper, "Optimized accurate auxiliary basis sets for RI-MP2 and RI-CC2 calculations for the atoms Rb to Rn" *Theor. Chem. Acc.* **2007**, *117*, 587-597. doi 10.1007/s00214-007-0250-5.
[26] A. V. Marenich, C. J. Cramer, D. G. Truhlar, "Universal Solvation Model Based on Solute Electron Density and on a Continuum Model of the Solvent Defined by the Bulk Dielectric Constant and Atomic Surface Tensions" *J. Phys. Chem. B* **2009**, *113*, 6378-6396. doi 10.1021/jp810292n.
[27] I. Sushko, S. Novotarskyi, R. Korner, A. K. Pandey, M. Rupp, W. Teetz, S. Brandmaier, A. Abdelaziz, V. V. Prokopenko, V. Y. Tanchuk, R. Todeschini, A. Varnek, G. Marcou, P. Ertl, V. Potemkin, M. Grishina, J. Gasteiger, C. Schwab, Baskin, II, V. A. Palyulin, E. V. Radchenko, W. J. Welsh, V. Kholodovych, D. Chekmarev, A. Cherkasov, J. Aires-de-Sousa, Q. Y. Zhang, A. Bender, F. Nigsch, L. Patiny, A. Williams, V. Tkachenko, I. V. Tetko, "Online chemical modeling environment (OCHEM): web platform for data storage, model development and publishing of chemical information" *J. Comput. Aided Mol. Des.* **2011**, *25*, 533-554. doi 10.1007/s10822-011-9440-2.
[28] E. V. Radchenko, A. S. Dyabina, V. A. Palyulin, N. S. Zefirov, "Prediction of human intestinal absorption of drug compounds" *Russ. Chem. Bull.* **2016**, *65*, 576-580. doi 10.1007/s11172-016-1340-0.
[29] A. S. Dyabina, E. V. Radchenko, V. A. Palyulin, N. S. Zefirov, "Prediction of blood-brain barrier permeability of organic compounds" *Dokl. Biochem. Biophys.* **2016**, *470*, 371-374. doi 10.1134/S1607672916050173.
[30] E. V. Radchenko, A. S. Dyabina, V. A. Palyulin, "Towards Deep Neural Network Models for the Prediction of the Blood-Brain Barrier Permeability for Diverse Organic Compounds" *Molecules* **2020**, *25*, 5901.doi 10.3390/molecules25245901.
[31] E. V. Radchenko, Y. A. Rulev, A. Y. Safanyaev, V. A. Palyulin, N. S. Zefirov, "Computer-aided estimation of the hERG-mediated cardiotoxicity risk of potential drug components" *Dokl. Biochem. Biophys.* **2017**, *473*, 128-131. doi 10.1134/S1607672917020107.
[32] ADMET Prediction Service. URL: http://qsar.chem.msu.ru/admet/
[33] G. R. Bickerton, G. V. Paolini, J. Besnard, S. Muresan, A. L. Hopkins, "Quantifying the chemical beauty of drugs" *Nat. Chem.* **2012**, *4*, 90-98. doi 10.1038/nchem.1243.
[34] RDKit: open-source cheminformatics software. URL: http://www.rdkit.org

Figure S1. NMR ^{1}H spectrum of 6-(1,3-dioxoisoindolin-2-yl)-*N*-(2,3,5,6,7,8-hexahydro-1*H*-cyclopenta[*b*]quinolin-9-yl)hexanamide

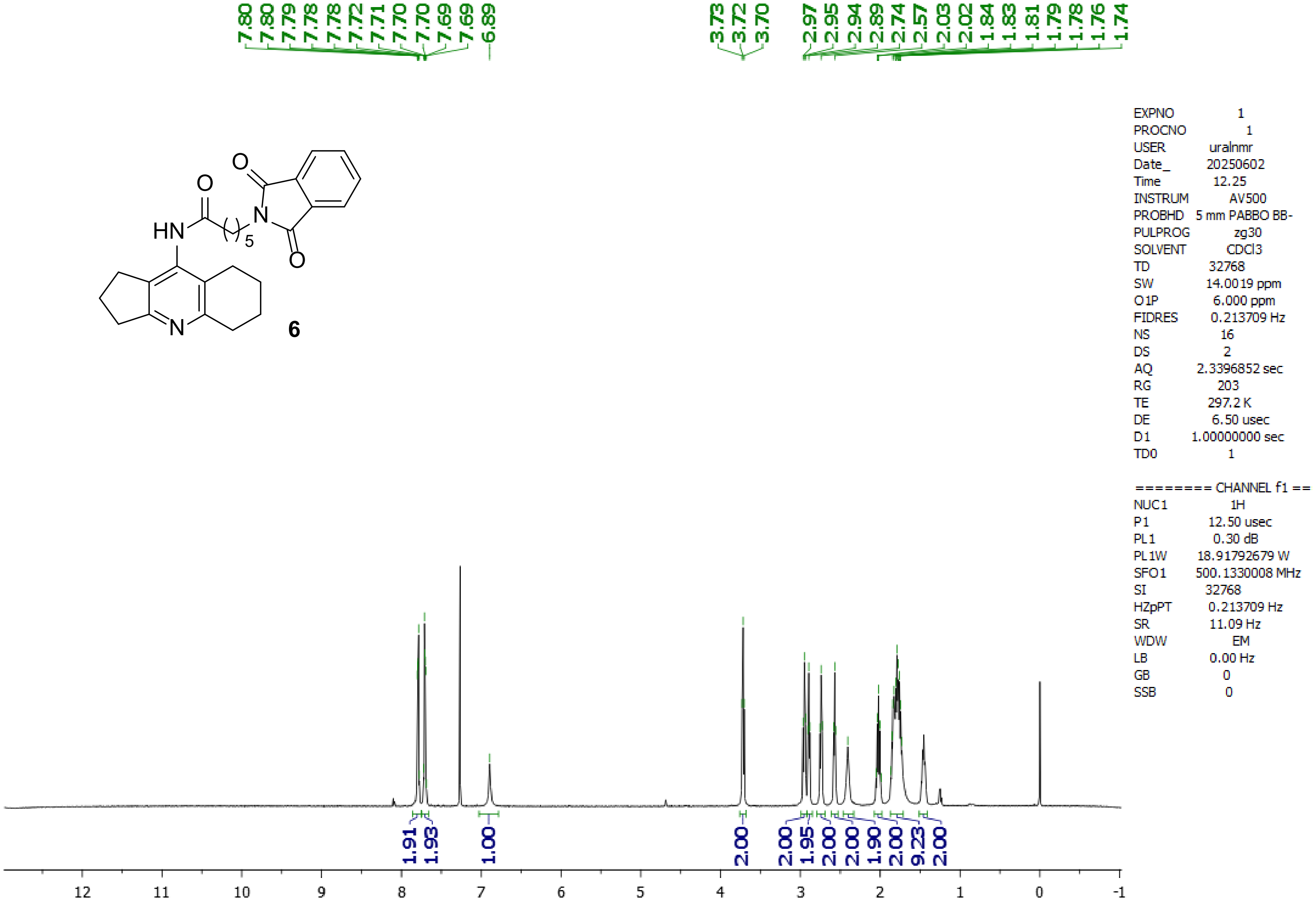

Figure S2. NMR ^{13}C spectrum of 6-(1,3-dioxoisoindolin-2-yl)-*N*-(2,3,5,6,7,8-hexahydro-1*H*-cyclopenta[*b*]quinolin-9-yl)hexanamide

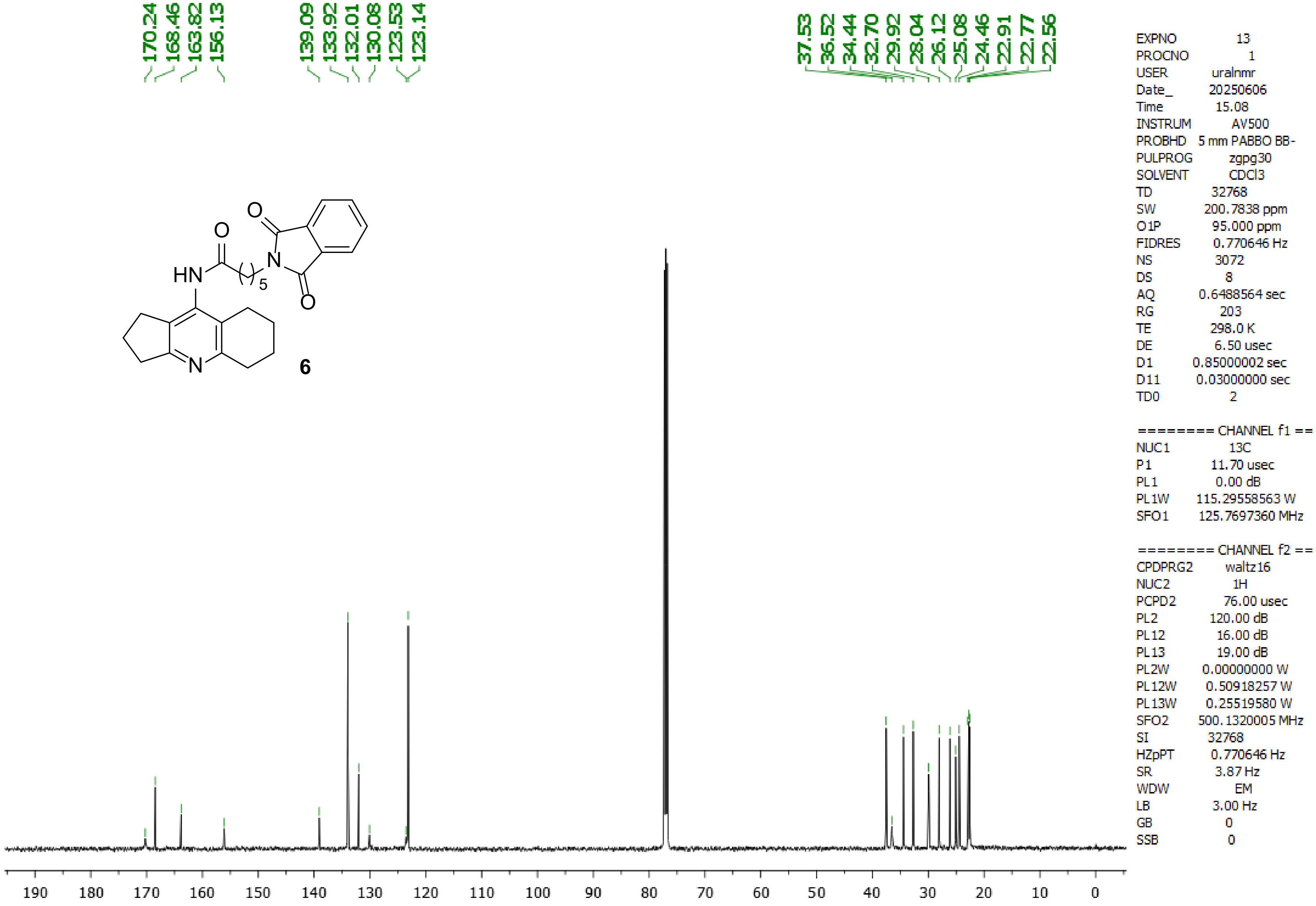

Figure S3. IR spectrum of 6-(1,3-dioxoisoindolin-2-yl)-*N*-(2,3,5,6,7,8-hexahydro-1*H*-cyclopenta[*b*]quinolin-9-yl)hexanamide

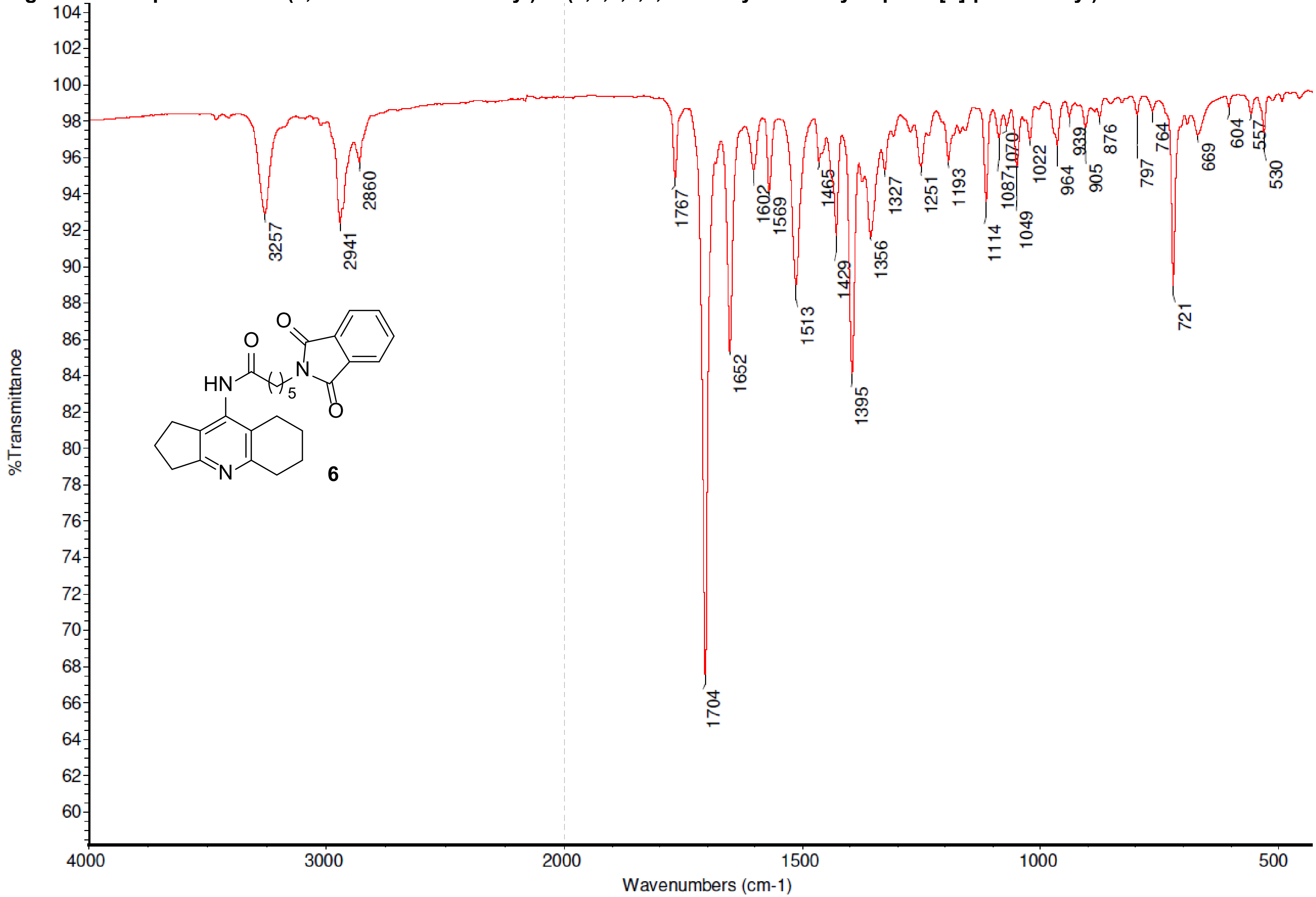

Figure S4. NMR ^{1}H spectrum of 6-amino-*N*-(2,3,5,6,7,8-hexahydro-1*H*-cyclopenta[*b*]quinolin-9-yl)hexanamide

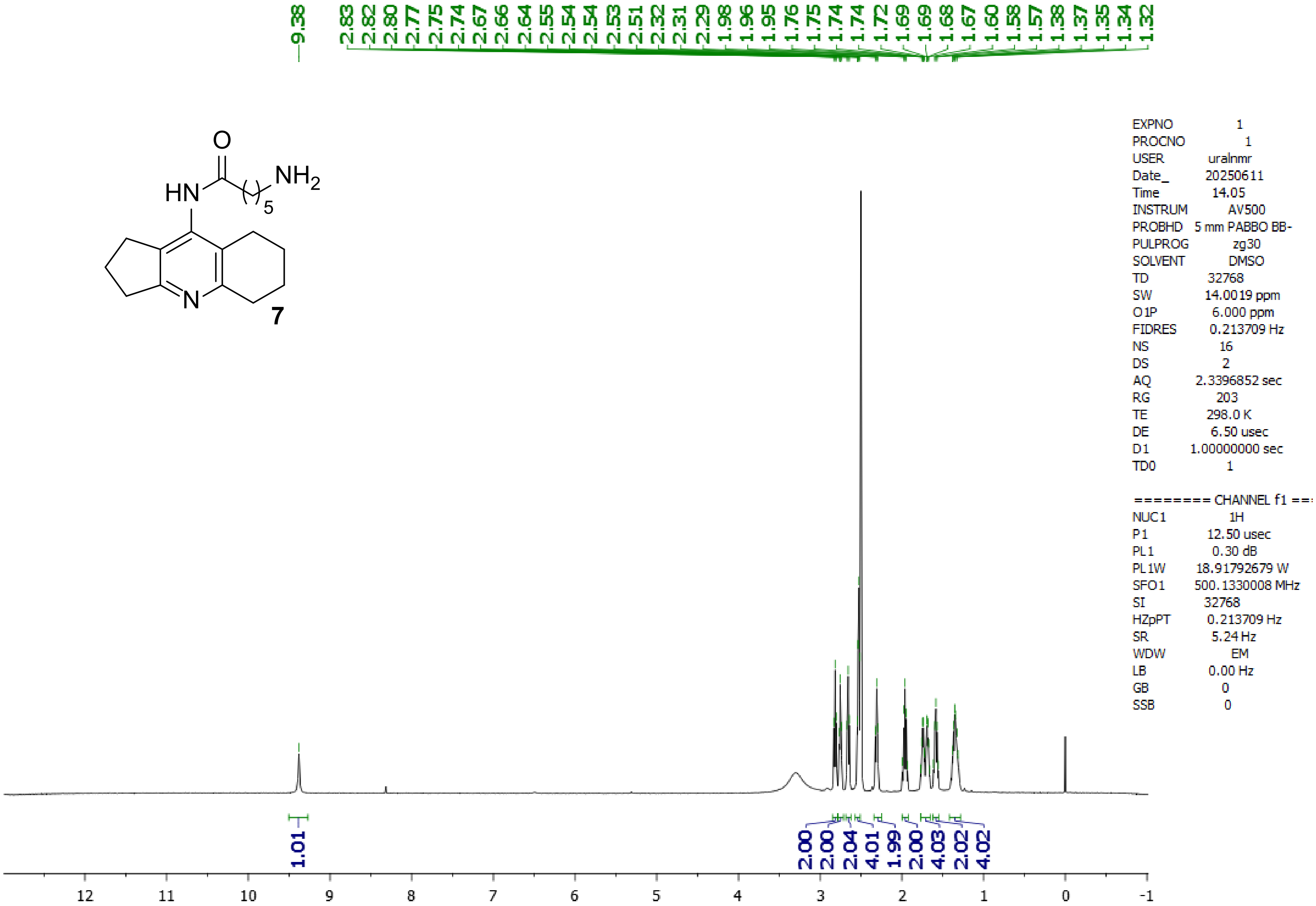

Figure S5. NMR ^{13}C spectrum of 6-amino-*N*-(2,3,5,6,7,8-hexahydro-1*H*-cyclopenta[*b*]quinolin-9-yl)hexanamide

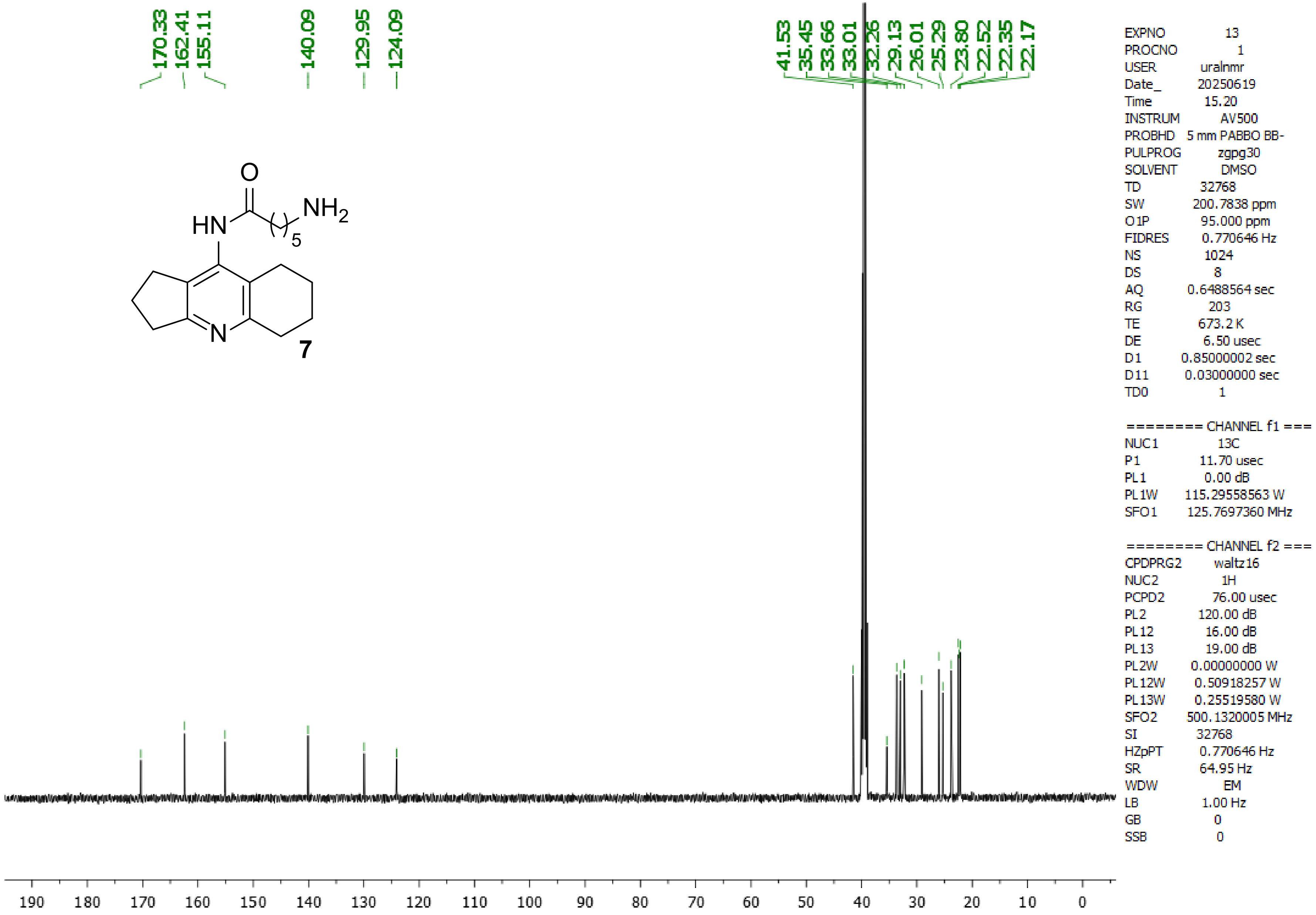

Figure S6. IR spectrum of 6-amino-*N*-(2,3,5,6,7,8-hexahydro-1*H*-cyclopenta[*b*]quinolin-9-yl)hexanamide

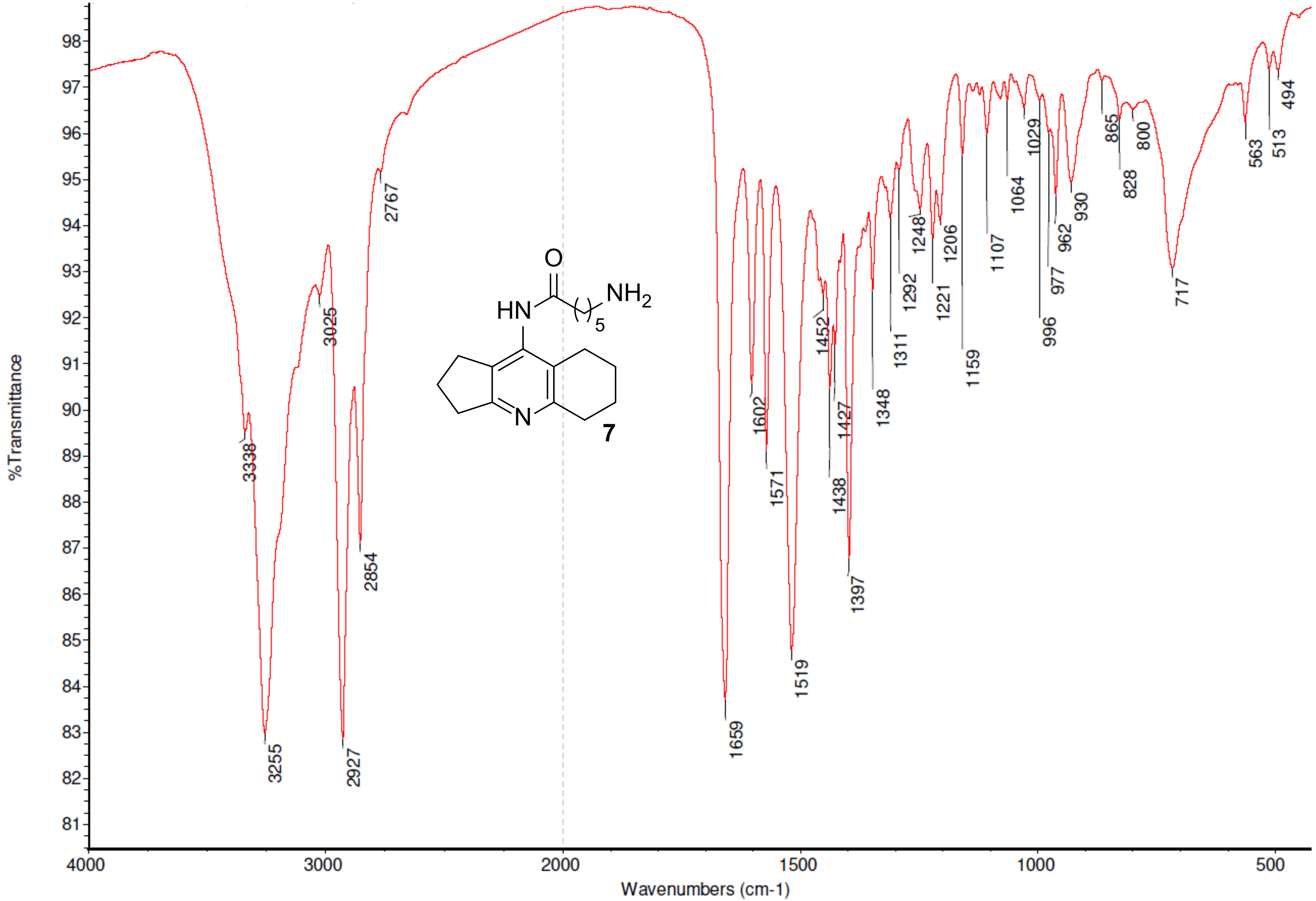

Figure S7. NMR 1H spectrum of N-(2,3,5,6,7,8-hexahydro-1H-cyclopenta[b]quinolin-9-yl)-6-((2-hydroxybenzylidene)amino)hexanamide

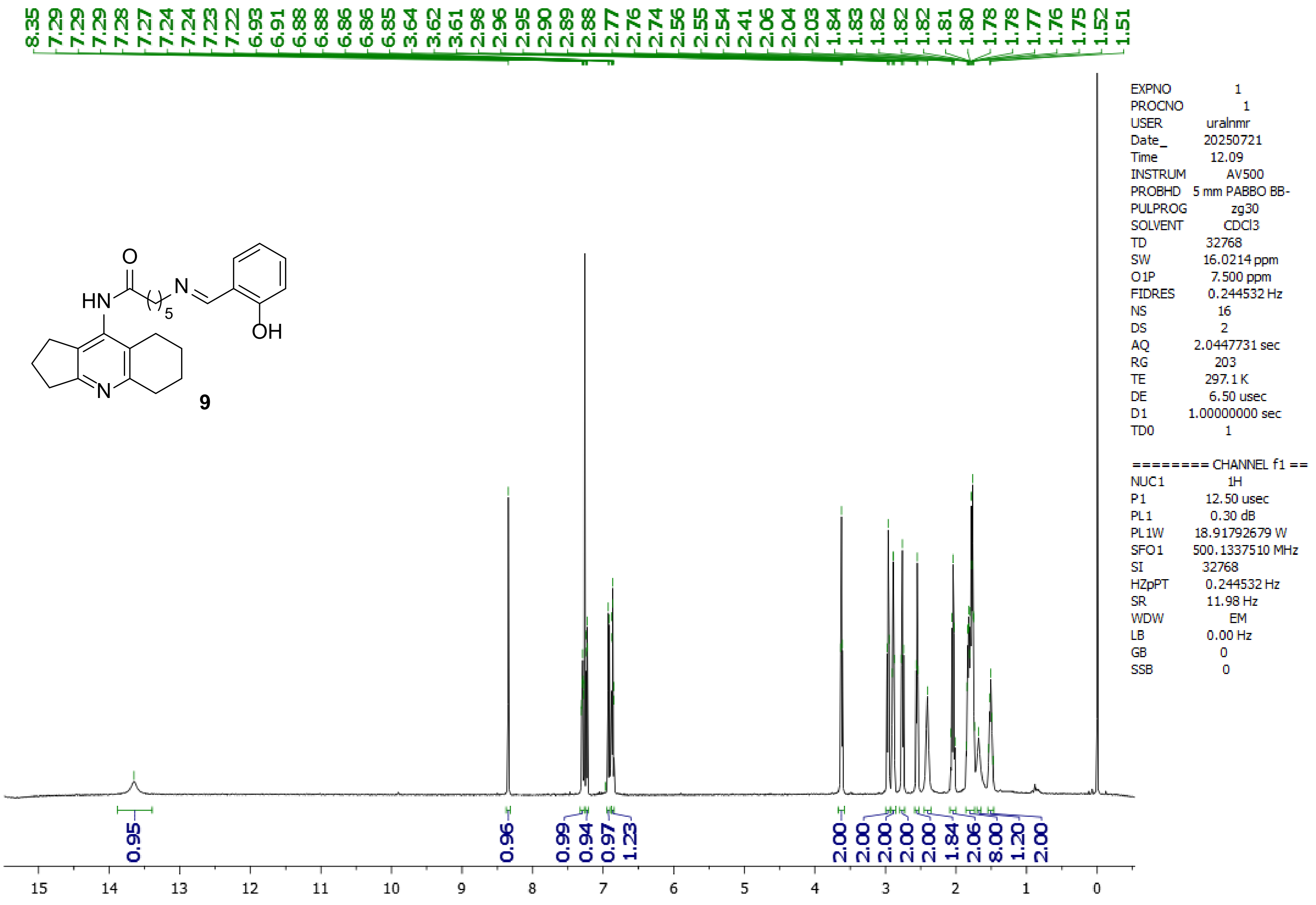

Figure S8. NMR ^{13}C spectrum of *N*-(2,3,5,6,7,8-hexahydro-1*H*-cyclopenta[*b*]quinolin-9-yl)-6-((2-hydroxybenzylidene)amino)hexanamide

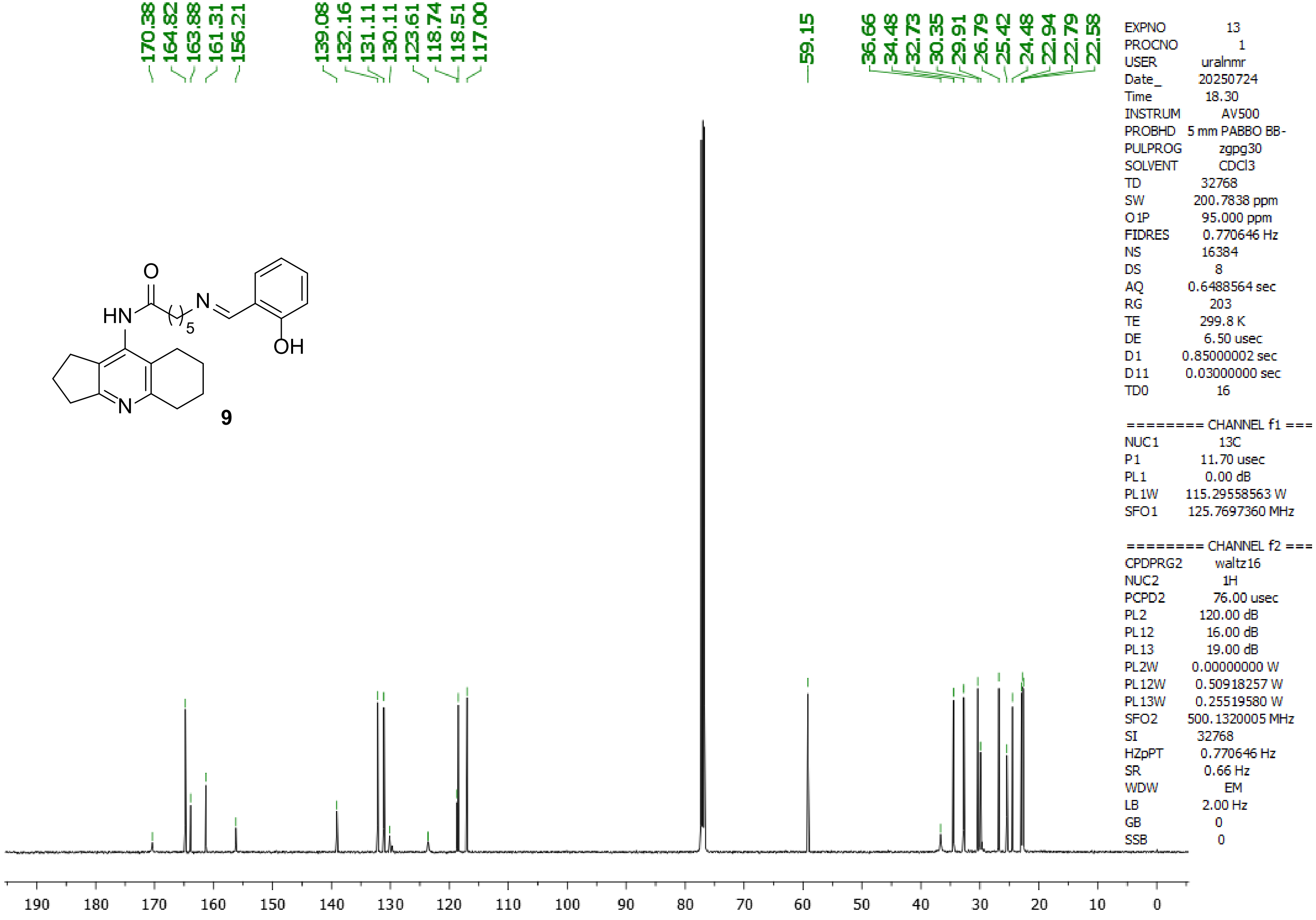

Figure S9. IR spectrum of *N*-(2,3,5,6,7,8-hexahydro-1*H*-cyclopenta[*b*]quinolin-9-yl)-6-((2-hydroxybenzylidene)amino)hexanamide

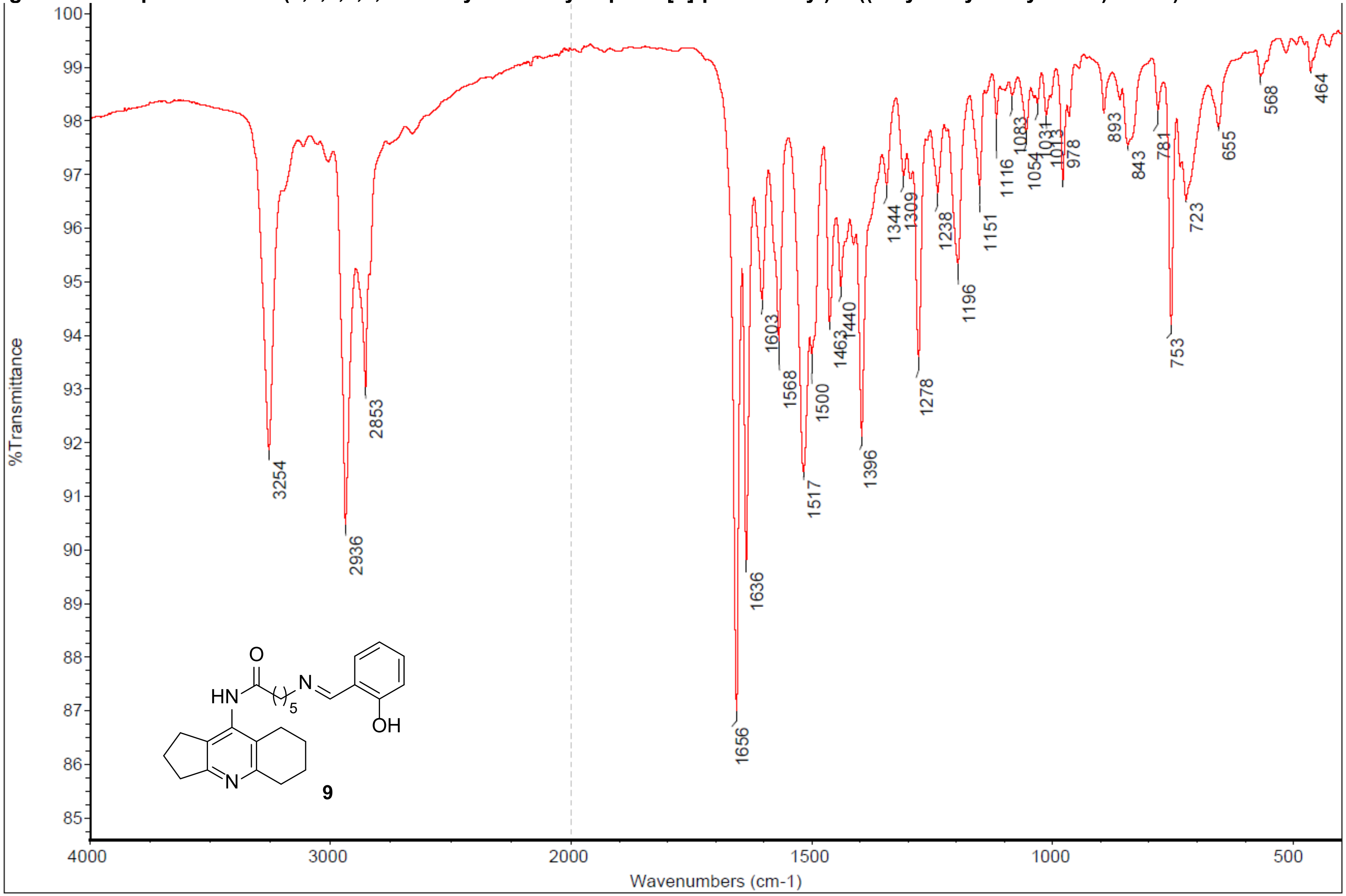

Figure S10. NMR ^{1}H spectrum of *N*-(2,3,5,6,7,8-hexahydro-1*H*-cyclopenta[*b*]quinolin-9-yl)-6-((2-hydroxybenzyl)amino)hexanamide

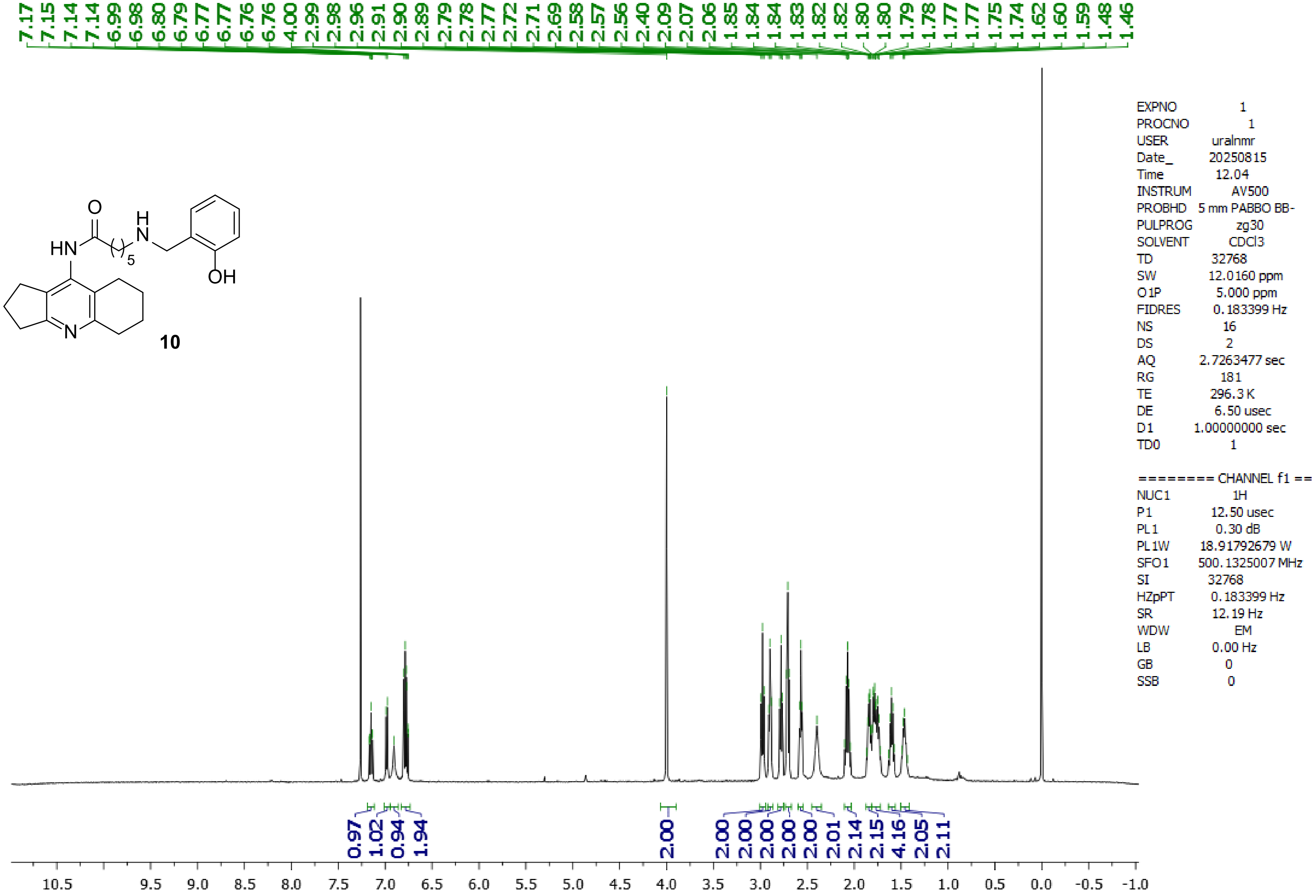

Figure S11. NMR ^{13}C spectrum of *N*-(2,3,5,6,7,8-hexahydro-1*H*-cyclopenta[b]quinolin-9-yl)-6-((2-hydroxybenzyl)amino)hexanamide

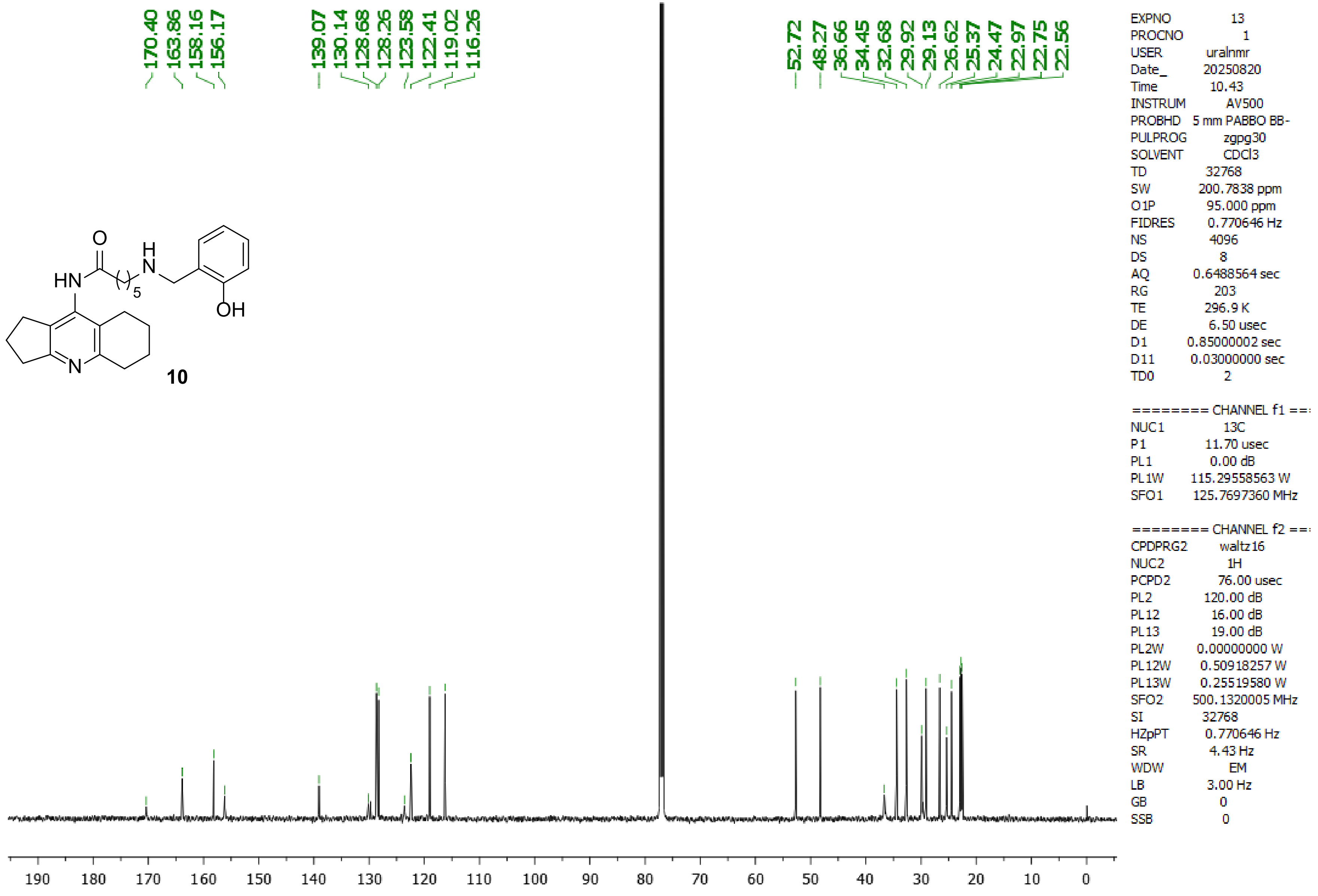

Figure S12. IR spectrum of *N*-(2,3,5,6,7,8-hexahydro-1*H*-cyclopenta[*b*]quinolin-9-yl)-6-((2-hydroxybenzyl)amino)hexanamide

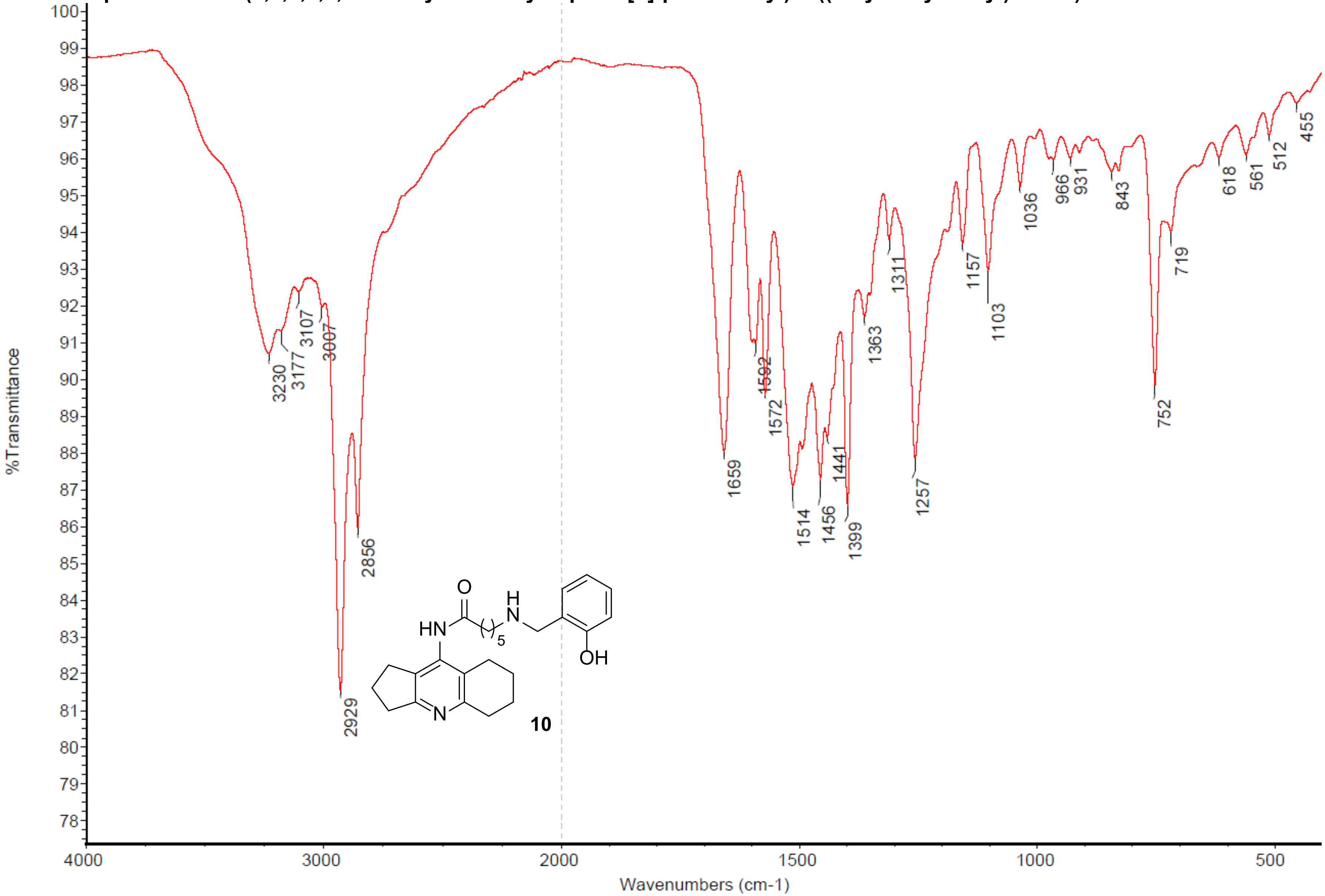

Figure S13. NMR ^{1}H spectrum of *N*-(2,3,5,6,7,8-hexahydro-1*H*-cyclopenta[*b*]quinolin-9-yl)hexanamide

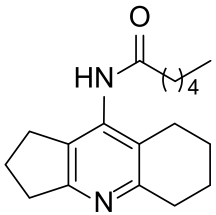

11

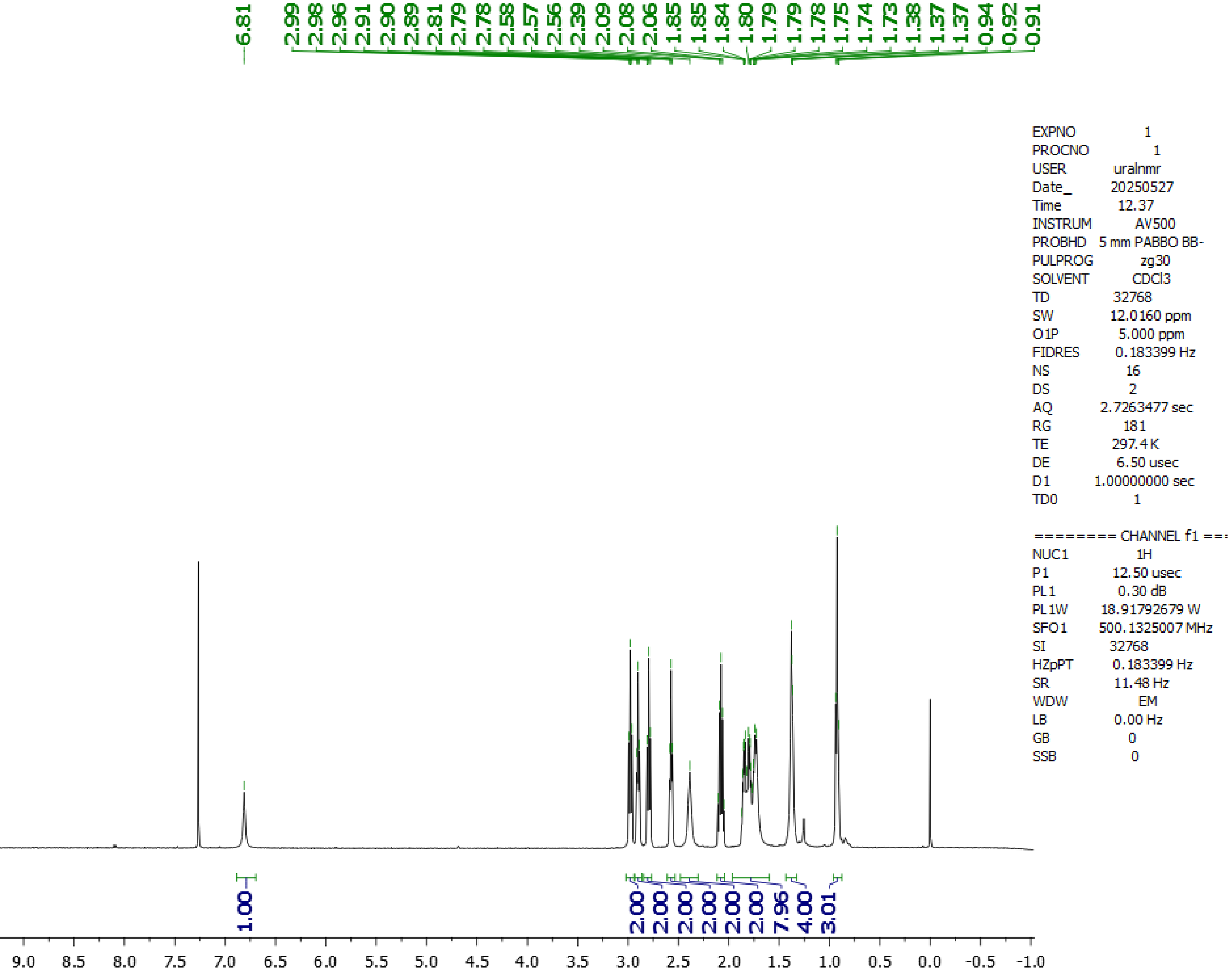

Figure S14. NMR ^{13}C spectrum of *N*-(2,3,5,6,7,8-hexahydro-1*H*-cyclopenta[*b*]quinolin-9-yl)hexanamide

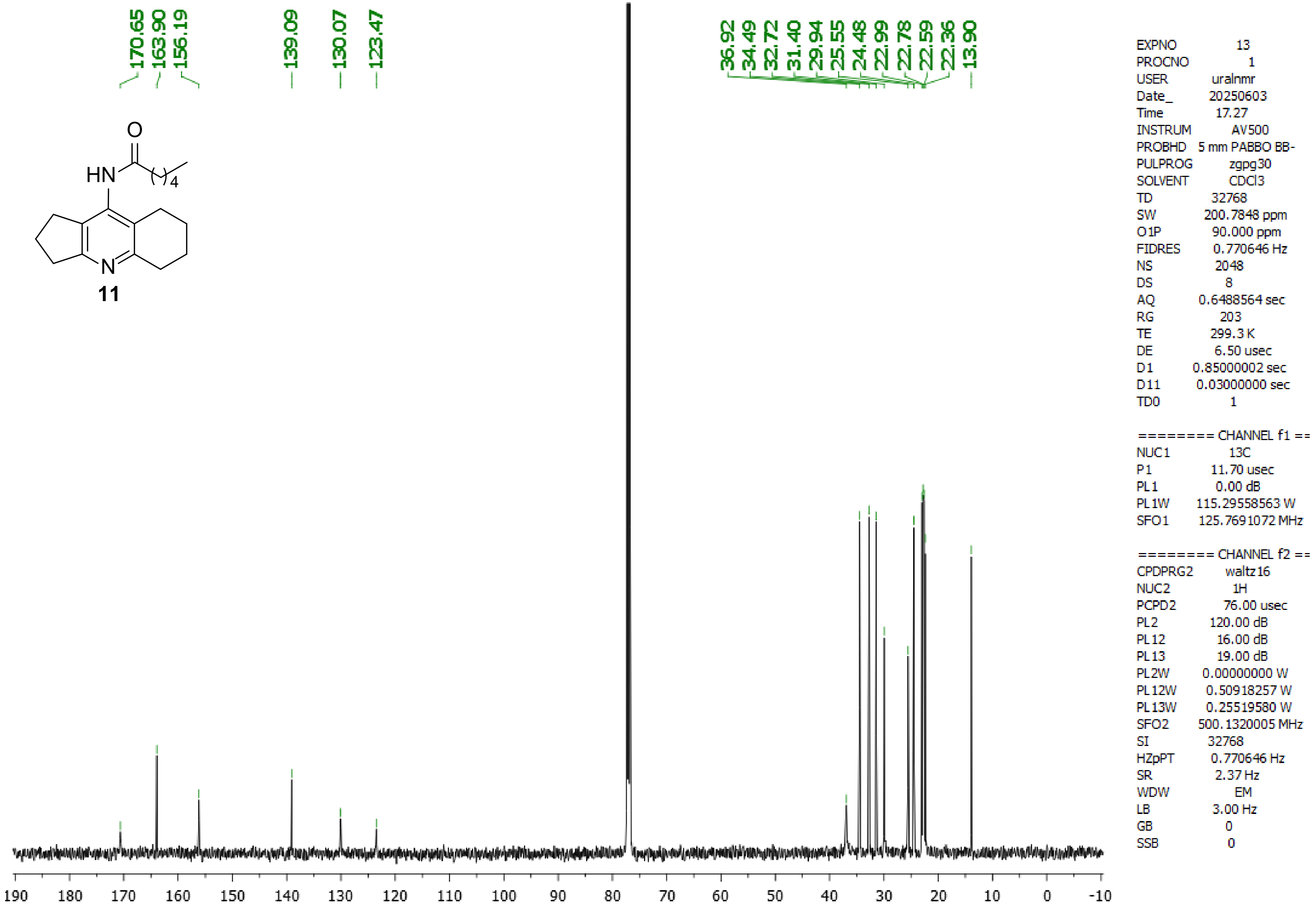

Figure S15. IR spectrum of *N*-(2,3,5,6,7,8-hexahydro-1*H*-cyclopenta[*b*]quinolin-9-yl)hexanamide

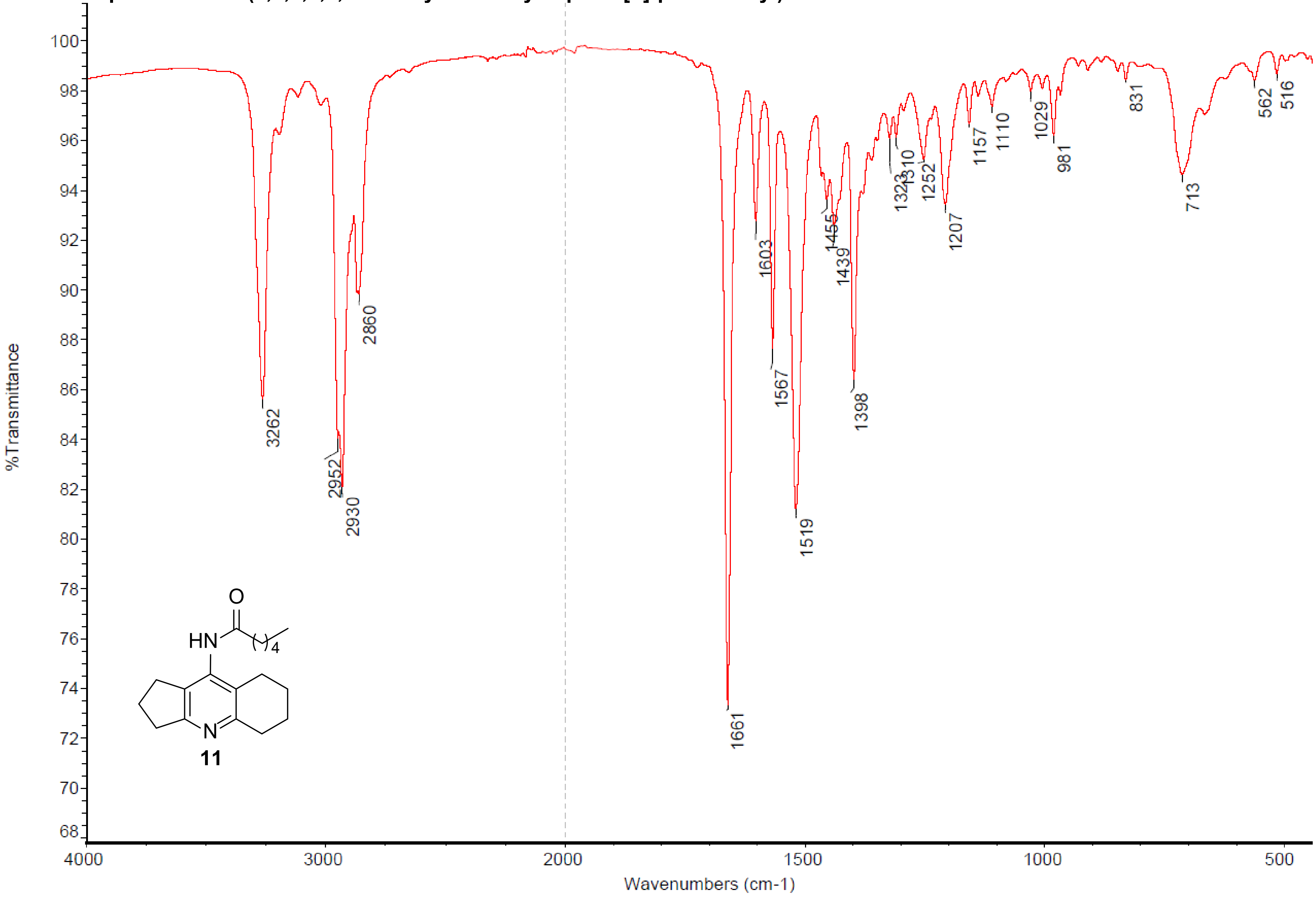

Figure S16. HRMS spectrum of 6-(1,3-dioxoisoindolin-2-yl)-*N*-(2,3,5,6,7,8-hexahydro-1*H*-cyclopenta[*b*]quinolin-9-yl)hexanamide

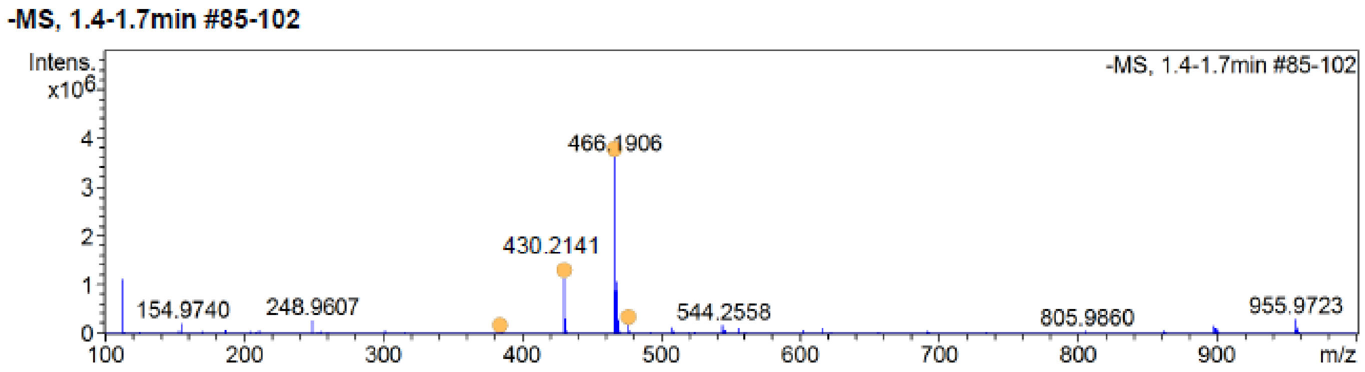

Figure S17. HRMS spectrum of *N*-(2,3,5,6,7,8-hexahydro-1*H*-cyclopenta[*b*]quinolin-9-yl)hexanamide

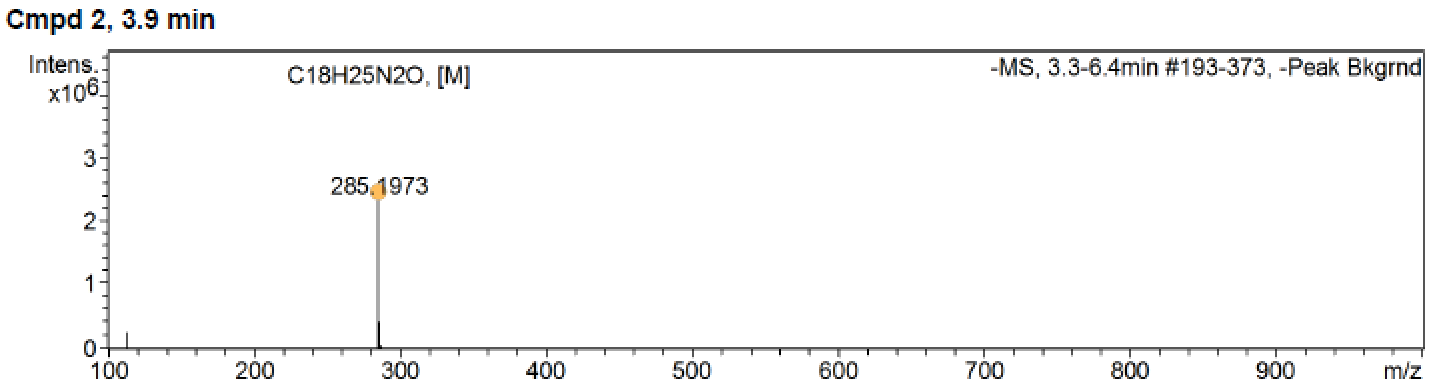

Supplement: Supplementary file 1 — Supplementary Material [file CMDC-21-e70392-s001.pdf]
